# Global insights into acetic acid resistance mechanisms and genetic stability of *Acetobacter pasteurianus* strains by comparative genomics

**DOI:** 10.1038/srep18330

**Published:** 2015-12-22

**Authors:** Bin Wang, Yanchun Shao, Tao Chen, Wanping Chen, Fusheng Chen

**Affiliations:** 1 Key Laboratory of Environment Correlative Dietology, Huazhong Agricultural University, Wuhan 430070, Hubei Province, P. R. China; 2 Hubei Provincial Cooperative Innovation Center of Industrial Fermentation, Hubei University of Technology, Wuhan 430068, Hubei Province, P. R. China; 3 College of Food Science and Technology, Huazhong Agricultural University, Wuhan 430070, Hubei Province, P. R. China

## Abstract

*Acetobacter pasteurianus* (*Ap*) CICC 20001 and CGMCC 1.41 are two acetic acid bacteria strains that, because of their strong abilities to produce and tolerate high concentrations of acetic acid, have been widely used to brew vinegar in China. To globally understand the fermentation characteristics, acid-tolerant mechanisms and genetic stabilities, their genomes were sequenced. Genomic comparisons with 9 other sequenced *Ap strains* revealed that their chromosomes were evolutionarily conserved, whereas the plasmids were unique compared with other *Ap* strains. Analysis of the acid-tolerant metabolic pathway at the genomic level indicated that the metabolism of some amino acids and the known mechanisms of acetic acid tolerance, might collaboratively contribute to acetic acid resistance in *Ap* strains. The balance of instability factors and stability factors in the genomes of *Ap* CICC 20001 and CGMCC 1.41 strains might be the basis for their genetic stability, consistent with their stable industrial performances. These observations provide important insights into the acid resistance mechanism and the genetic stability of *Ap* strains and lay a foundation for future genetic manipulation and engineering of these two strains.

Acetic acid bacteria (AAB) are a group of gram-negative or gram-variable obligate aerobic bacteria within the class alpha-proteobacteria^1^ and have been isolated from a variety of natural sites, including fermented food^2^,^3^,^4^,^5^, plant organs^6^,^7^, animal organs^8^ and soil^9^,^10^. Since *Acetobacter* (*A.*) was recognized in 1898^11^, 17 genera consisting of approximately 88 species in total, have been recorded in the AAB group^12^,^13^,^14^,^15^. Among them, *Acetobacter* and *Komagataeibacter* (*K.,* formerly classified as *Gluconacetobacter*^16^) are the main AAB genera that have been widely used to brew vinegar^17^,^18^. For example, *K. europaeus* strains contribute to fruit and alcohol vinegars by liquid-state fermentation in Europe^19^,^20^,^21^, while *A. pasteurianus* (*Ap*) strains are mainly used to brew cereal vinegars by liquid-state or solid-state fermentation in Asia, especially in China and Japan^17^,^22^,^23^.

In the vinegar industry, instabilities in the ability of AAB strains to produce and tolerate acetic acid may be the most critical factors leading to instability in acetic acid production. Instabilities of fermentative and genetic characteristics of *K. europaeus* have been well-recognized^24^,^25^. However, only a few papers related to the instabilities of the fermentative and genetic characteristics in *Ap* have been published. Azuma *et al.* found that there were a large amount of transposons and some hyper-mutable tandem repeats in the genomes of *Ap* IFO 3283 substrains, indicating their genetic instability^26^, while Gullo *et al.* proved that *Ap* AB0220 maintained the quondam features, such as ethanol oxidation, acetate assimilation, phenotypic traits and 16S rRNA gene sequences, after 9 years of preservation^27^. In China, *Ap* CICC 20001 (once named *Huniang* 1.01) and CGMCC 1.41 (once named AS1.41), which were isolated from a vinegar factory in Dandong by the Shanghai Institute of Brewing and bred by the Institute of Microbiology Chinese Academy of Sciences, respectively, may be the earliest screened AAB isolates and are still widely used to brew vinegar by solid-state and liquid-state fermentation, displaying high stabilities in acetic acid production^28^,^29^,^30^,^31^.

In addition to genetic instability, the mechanisms related to acetic acid resistance in AAB have been a hot research topic. Although some mechanisms conferring acetic acid resistance in AAB, such as acetate assimilation, transportation systems, cell membrane composition, and stress proteins expression, have been reviewed^15^,^32^, the mechanisms of acetic acid resistance in AAB remain unclear. *Escherichia* (*E.*) *coli* is one of the microorganisms whose acid resistance mechanisms have been extensively studied and thoroughly understood. In *E. coli*, the metabolic responses, the chloride transporters, the oxidative system, the cyclopropane fatty acid, the arginine-dependent system, the glutamate-dependent system and the lysine-dependent system are elegantly regulated systems that permit *E. coli* to survive when a nurturing environment at pH 7 declines sharply to a harsh pH 2 milieu^33^,^34^,^35^,^36^,^37^. Furthermore, other mechanisms, such as arginine deiminase pathway and urease system also have been proved to confer acid resistance in bacteria^38^,^39^,^40^,^41^. Acid resistance mechanisms in *E. coli* and other bacteria may provide a reference for investigating acetic acid resistance mechanisms in AAB using comparative genomics.

The combination of whole genome sequencing and subsequent genomic analysis^42^,^43^,^44^,^45^, is an effective method to investigate gene functions, genetic information and biological characteristics, which may contribute to the dissection of product and genetic stability in *Ap*. Genomic analysis can be used to construct the metabolic blueprint for *Ap*, by comparisons with the known mechanisms conferring acid tolerance and the known metabolic pathways in microorganisms. However, as far as we know, no study has been conducted to investigate acid tolerance in microorganisms by analyzing their overall metabolic pathway.

In this study, the fermentation characteristics of *Ap* CICC 20001 and CGMCC 1.41 were investigated. Then, to globally understand their fermentation characteristics, as well as the mechanisms conferring acetic acid resistance, the complete genomes of *Ap* CICC 20001 and CGMCC 1.41 were sequenced and analyzed. Comparisons of *Ap* CICC 20001 and CGMCC 1.41 with other sequenced *Ap* strains revealed differences among *Ap* strains. Furthermore, an integral understanding of the molecular mechanisms underlying their acetic acid tolerance has been undertaken by arranging a metabolic blueprint related to acetic acid resistance in *Ap* strains, which may lead to detailed information for improving their abilities to produce and tolerate acetic acid during vinegar fermentation.

## Results

### Comparison of fermentation characteristics of *Ap* CICC 20001 and CGMCC 1.41

Both *Ap* CICC 20001 and CGMCC 1.41 have been widely used to brew vinegar in China. However, they display different fermentation characteristics in vinegar industry, such as acetic acid resistance and production. To investigate the differences, *Ap* CICC 20001 and CGMCC1.41 were inoculated into a modified GYP medium containing different concentrations of ethanol and acetic acid.

#### The effects of initial acetic acid concentration on acetic acid production in Ap CICC 20001 and CGMCC 1.41

**Figure 1:**
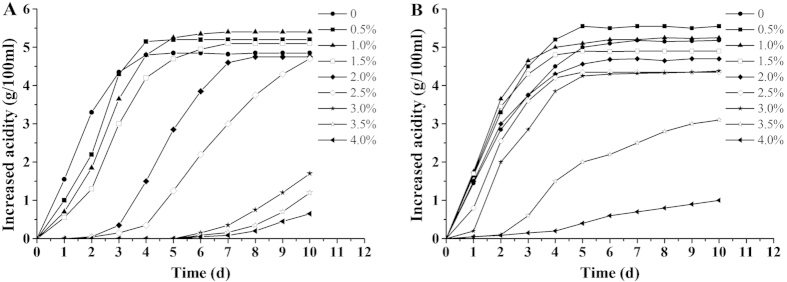
Effects of initial acetic acid concentration on fermentation in *Ap* CICC 20001 (A) and CGMCC 1.41 (B). They were cultivated in a 250 ml Erlenmeyer flask containing 50 ml GYP medium with 6% ethanol and 0, 0.5%, 1%, 1.5%, 2%, 2.5%, 3%, 3.5% or 4% acetic acid.

To investigate the effects of the initial amount of acetic acid on acetic acid production, *Ap* CICC 20001 and CGMCC 1.41 were inoculated into modified GYP medium containing 6% ethanol and different concentrations of acetic acid (Fig. 1). The results showed that a low initial concentration of acetic acid (0.5% ~ 1%) could promote acetic acid fermentation in *Ap* CICC 20001 and CGMCC 1.41. *Ap* CGMCC 1.41 and CICC 20001 produced the highest concentration of acetic acid at 6% ethanol, with initial concentrations of 0.5% and 1% acetic acid, respectively. However, the high initial acid concentration would also inhibit the fermentation process. The lengths of the lag phase in *Ap* CICC 20001 and CGMCC 1.41 increased considerably when the initial concentration of acetic acid was greater than 1.5% and 3%, respectively (Fig. 1).

#### Ap CGMCC 1.41 produced a higher concentration of acetic acid than Ap CICC 20001

**Figure 2:**
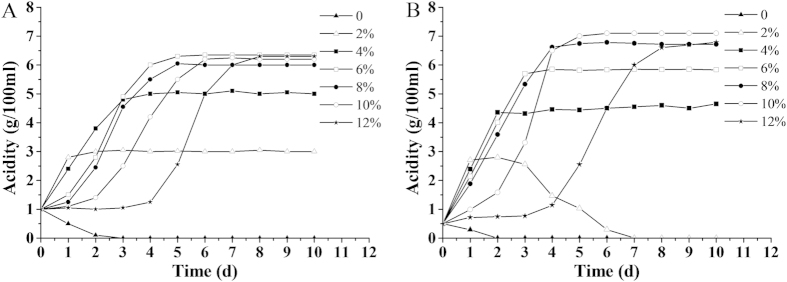
Production of acetic acid in *Ap* CICC 20001 (A) and CGMCC 1.41 (B). They were cultivated in a 250 ml Erlenmeyer flask containing 50 ml GYP medium with 1% acetic acid in *Ap* CICC 20001 and 0.5% acetic acid in *Ap* CGMCC 1.41, and 0, 2%, 4%, 6%, 8%, 10% or 12% ethanol.

*Ap* CICC 20001 and CGMCC 1.41 were inoculated into modified GYP medium containing a range of concentrations of ethanol and optimized concentrations of acetic acid (1% in *Ap* CICC 20001 and 0.5% in *Ap* CGMCC 1.41) (Fig. 2). *Ap* CICC 20001 yields the maximum acetic acid (6.35%) in the GYP medium with 6% ethanol and 1% initial acetic acid, while *Ap* CGMCC 1.41 produces the maximum acetic acid (7.15%) in GYP medium with 10% ethanol and 0.5% initial acetic acid. There is a positive relationship between the initial ethanol concentration and the acetic acid produced within a certain range during fermentation by *Ap* CICC 20001 and CGMCC 1.41. Furthermore, high levels of ethanol also inhibit acetic acid production. The lengths of lag phase in *Ap* CICC 20001 and CGMCC 1.41 increase considerably, when the initial concentrations of ethanol are greater than 6% and 10%, respectively. *Ap* CGMCC 1.41 also exhibits an obvious peroxidation in the medium with 2% ethanol and 0.5% acetic acid. However, *Ap* CICC 20001 has a weak peroxidation ability.

### Features of the *Ap* CICC 20001 and CGMCC 1.41 genomes

To gain insights into the fermentation characteristics of these two *Ap* strains from genomic analysis, *Ap* CICC 20001 and CGMCC 1.41 cells were subjected to single molecule real time (SMRT) sequencing using a PacBio RS instrument. Previously, genomic sequencing of 9 *Ap* strains, including *Ap* 386B, 7 IFO 3283 substrains and 1 IFO 3283 mutant IFO 3283-01-42C, had been completed and published. Among the *Ap* strains, 386B was isolated from a spontaneous cocoa bean heap fermentation in Ghana^4^, while IFO 3283 was isolated from a pellicle formed on the surface during fermentation of traditional Japanese rice vinegar in 1954^26^. Seven substrains of IFO 3283 were isolated from IFO 3283 after it had been stored for more than 30 years, and the mutant substrain IFO 3283-01-42C was trained and isolated. In this study, we present genomic sequencing of *Ap* CICC 20001 and CGMCC 1.41 and the genomic comparison of these strains with 9 other *Ap* strains. The genome sequences of 11 *Ap* strains have been published.

#### Plasmids in Ap CICC 20001 may play a greater role in metabolism than they do in Ap CGMCC 1.41

The chromosome of *Ap* CICC 20001 contains 2865612 bp, with a 52.94% G + C content, 3006 predicted coding sequences (CDSs), 118 tRNAs, 15 mobile elements and 2 prophages, while that of *Ap* CGMCC 1.41 contains 2928931 bp, with a 52.95% G + C content, 3012 CDSs, 111 rRNAs, 56 mobile elements and 2 prophages (Table 1). Their chromosomes are analogous, but their plasmids are completely different from each other, not only in encoded genes but also in the number of plasmids. Specific functions are assigned to 67.6% (2450 genes) of the total of 3623 protein-coding genes in *Ap* CICC 20001 and 69.2% (2248 genes) of the total of 3250 protein-coding genes in *Ap* CGMCC 1.41, and the remaining genes are hypothetical genes.

**Figure 3:**
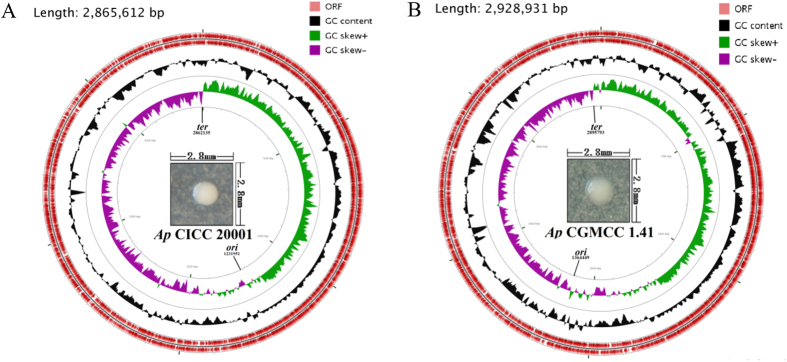
Chromosomal features of *Ap* CICC 20001 and CGMCC 1.41. From outer to inner circles, the first, second, third and fourth circle represent the positive strand ORF, the negative strand ORF, the GC content and the GC skew, respectively. The middle figure shows a colony grown on a GYC medium plate for 5 d.

The GC skew [(G − C)/(G + C)] and the cumulative GC skew have proven to be useful as indicators of the DNA leading strand, lagging strand, replication origin, and replication terminal^46^. The putative replication origin (*ori*) and terminal (*ter*) of the chromosomes were predicted, based on the GC skew and the cumulative GC skew. The general chromosome features of *Ap* CICC 20001 and CGMCC 1.41, including *ori*, *ter* and G + C content, as well as colony characteristics, are shown in Fig. 3. The *ori* and *ter* of *Ap* CICC 20001 may be located at 1231951 bp and 2862135 bp, respectively, while those of *Ap* CGMCC 1.41 may be located at 1364449 bp and 2895793 bp, respectively. Colonies of *Ap* CICC 20001 and CGMCC 1.41 that are cultivated on GYC medium for 5 d have their own features. A colony of *Ap* CICC 20001 (approximately 0.8 mm) is smaller than one of *Ap* CGMCC 1.41 (approximately 1.2 mm). Furthermore, both *Ap* CICC 20001 and CGMCC 1.41 display highly similar orthologous groups (Supplementary Fig. S1).

#### Chromosomes of Ap CICC 20001 and CGMCC 1.41 display a high homology with other Ap strains

**Figure 4:**
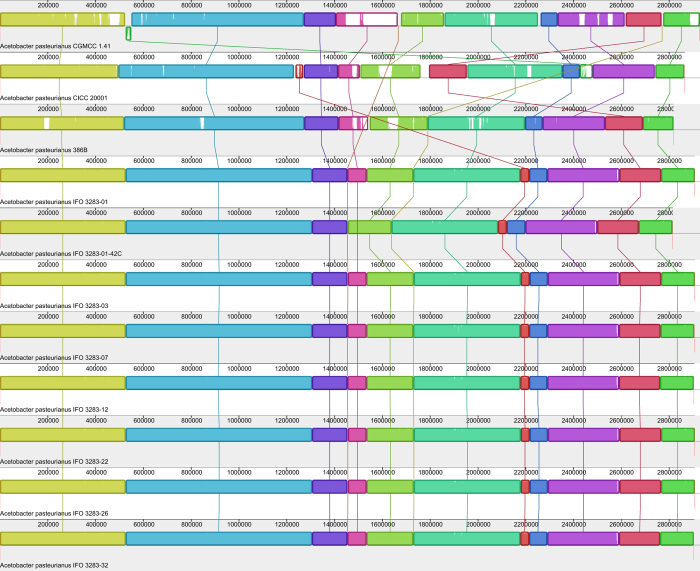
The locations of orthologs between chromosomes of 11 *Ap* strains. The lines represent (from top to bottom): line 1, chromosome of *Ap* CGMCC 1.41; line 2, chromosome of *Ap* CICC 20001; line 3, chromosome of *Ap* 386B; line 4, chromosome of *Ap* IFO 3283-01; line 5, chromosome of *Ap* IFO 3283-01-42C; line 6, chromosome of *Ap* IFO 3283-03; line 7, chromosome of *Ap* IFO 3283-07; line 8, chromosome of *Ap* IFO 3283-12; line 9, chromosome of *Ap* IFO 3283-22; line 10, chromosome of *Ap* IFO 3283-26; and line 11, chromosome of *Ap* IFO 3283-32. Fragments of the same color represent orthologous regions in different *Ap* strains.

To explore the relationships among the sequenced *Ap* strains, including *Ap* CICC 20001, CGMCC 1.41 and the previously sequenced *Ap* species, their chromosomes were compared using Mauve software. *Ap* CICC 20001 and CGMCC 1.41 share a high degree of homology with the 9 published *Ap* strains. However, the chromosome sequence of *Ap* CGMCC 1.41 is slightly longer than that of *Ap* CICC 20001 and the IFO 3283 substrains (Fig. 4). Although there are high degrees of similarity among the 11 *Ap* strains, there are also deletions, amplifications, insertions, inversions and translocations. Compared to *Ap* IFO 3283 substrains and 386B, some DNA fragments are missing.

#### The plasmids of Ap CICC 20001 and CGMCC 1.41 are unique

**Figure 5:**
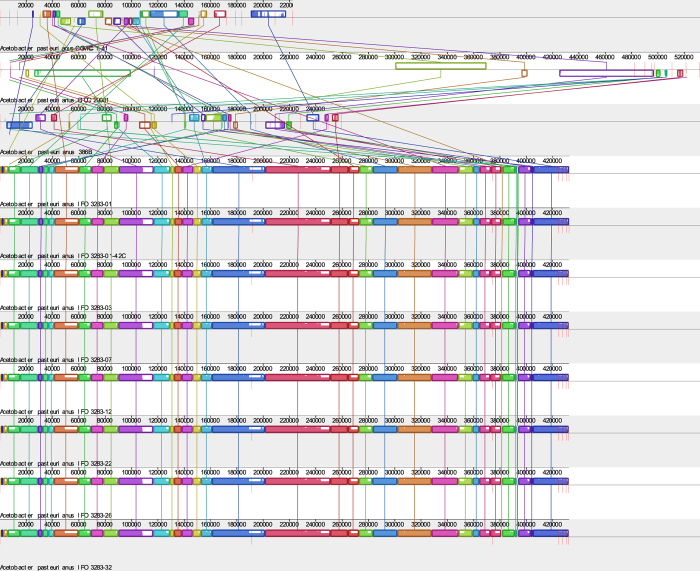
The locations of orthologs between plasmids of 11 *Ap* strains. The lines represent (from top to bottom): line 1, plasmids of *Ap* CGMCC 1.41; line 2, plasmids of *Ap* CICC 20001; line 3, plasmids of *Ap* 386B; line 4, plasmids of *Ap* IFO 3283-01; line 5, plasmids of *Ap* IFO 3283-01-42C; line 6, plasmids of *Ap* IFO 3283-03; line 7, plasmids of *Ap* IFO 3283-07; line 8, plasmids of *Ap* IFO 3283-12; line 9, plasmids of *Ap* IFO 3283-22; line 10, plasmids of *Ap* IFO 3283-26; and line 11, plasmids of *Ap* IFO 3283-32. The red vertical lines on each line represent the edges of different plasmids.

To compare the plasmids of different *Ap* strains, their plasmids were integrated severally and aligned using Mauve 2.3.1 (Fig. 5). The results show that plasmids have specific characteristics of *Ap* strains from different sources. Compared to *Ap* IFO 3283 substrains and 386B, CICC 20001 and CGMCC 1.41 possess few similar genotypes. The plasmids of *Ap* CICC 20001, CGMCC 1.41, 386B and IFO 3283 are largely different. Most notably, *Ap* CICC 20001 has a large plasmid of approximately 474484 bp, which has almost no homology to plasmids from other *Ap* strains. This plasmid may play a key role in protecting the genomic integrity of *Ap* CICC 20001 because it contains 3 CRISPR (clustered regularly interspaced short palindromic repeats) elements, as discussed later. Furthermore, it appears that similar to the chromosome, the plasmids display genetic stability after storage for more than 30 years. Plasmids of all IFO 3283 substrains also have very similar genetic sequences. The relative positions of the genes in the plasmids of the substrains are identical, with the exception of two transposases, APA01_40012 in IFO 3283-01-42C and APA42C_40012 in IFO 3283-01^26^, which were inserted into related plasmids without interrupting the coding sequence of a gene. Among *Ap* IFO 3283 substrains, IFO 3283-32 possesses the least variation in its chromosome and plasmids; therefore, *Ap* IFO 3283-32 is used to represent all *Ap* IFO 3283 substrains in subsequent analyses.

#### More than 100 essential genes support the survival of Ap strains

Essential genes are thought to be critical for the survival of organisms. Essential genes in *Ap* strains were predicted using ZCURVE 3.0 software. *Ap* IFO 3283 substrains have extremely similar chromosomes with the same essential genes, and *Ap* IFO 3283-32 are used as a representative of *Ap* IFO 3283 substrains. The distribution of essential genes in chromosomes of *Ap* IFO 3283-32, 386B, CICC 20001 and CGMCC 1.41 are shown in Supplementary Fig. S2. The essential genes pervade chromosomes. In each chromosome, there is more than one cluster of essential genes and some large regions (longer than 300 kb) without essential genes. These essential genes encode proteins that primarily participate in maintaining basic cellular structure, replicating DNA, translating genes into proteins, mediating transport processes into and out of the cell, and maintaining central metabolism.

#### Amino acid composition among Ap strains is similar

**Figure 6:**
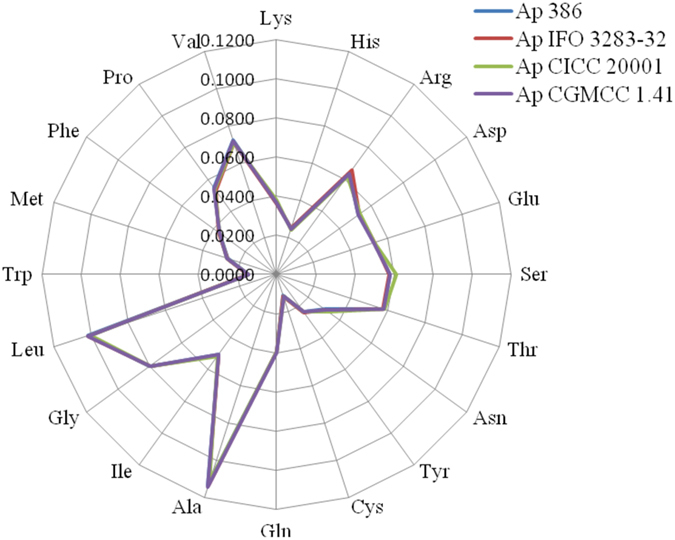
Amino acid components in *Ap* 386B, IFO 3283-32, CICC 20001 and CGMCC 1.41.

*Ap* IFO 3283-32 is characterized to be a representative of IFO 3283 substrains, which are highly similar in their gene distributions and coded proteins. Amino acid components from *Ap* IFO 3283-32, 386B, CICC 20001 and CICC 1.41 were computed. Amino acid components in different *Ap* species are highly similar, although *Ap* strains share a high homology in the chromosome and almost no similarity in their plasmids (Fig. 6). All of them have a high concentration of alanine, leucine and glycine. *Ap* IFO 3283-32 and CICC 20001 have higher levels of arginine and serine, respectively.

### Plausible mechanisms conferring acetic acid resistance and related metabolism in *Ap*

Because *Ap* strains have highly similar genomes, in the presence of a high concentration of acetic acid, they may also exhibit similar mechanisms conferring acetic acid resistance. Some mechanisms related to acid resistance in AAB and *E. coli*, such as transportation systems, stress proteins, metabolic responses, chloride transporters, arginine-dependent system, glutamate-dependent system and lysine-dependent system, have been discovered^15^,^33^,^35^,^47^,^48^. The comparison of protein-coding genes that contribute to acid resistance can be used to discover mechanisms conferring acetic acid resistance and to construct a metabolic blueprint related to acetic acid tolerance in *Ap*.

#### Comparative genomic analysis emphasizes the roles of PQQ-ADH in acetic acid production and resistance in AAB

Pyrroloquinoline quinine dependent alcohol dehydrogenase (PQQ-ADH) and aldehyde dehydrogenase (PQQ-ALDH) are membrane-bound enzymes in AAB, which catalyze the oxidation of alcohol to aldehyde and aldehyde to acetic acid, respectively, producing abundant extracellular acetic acid^49^. Both PQQ-ADH and PQQ-ALDH play a key role in the respiratory chain in *Ap* CGMCC 1.41, CICC 20001 and other AAB^31^,^50^. Trcek *et al.* demonstrated that PQQ-ADH is involved in acetic acid resistance by comparing the enzymatic activities of PQQ-ADH from the AAB strains with different acetic acid production abilities^21^. *K. europaeus*, *K. oboediens* and *Ap* produce a high concentration of acetic acid of up to 18%, 8% and 6%, respectively^51^,^52^. In combination with their gradient abilities to produce acetic acid, the gene number of ADH and ALDH may further reveal the relation between ADH, as well as ALDH, and acetic acid tolerance.

To emphasize the effects of ADHs and ALDHs on producing a high level of acetic acid, genes coding ADH and ALDH in *Ap* CICC 20001, *Ap* CGMCC 1.41, *Ap* 386B, *Ap* IFO 3283-32, *K. europaeus* 5P3, and *K. oboediens* 174Bp2 were investigated (Table 2). Membrane-bound ADHs (Supplementary Data S1) and ALDHs (Supplementary Data S2), together with their topologies (Supplementary Fig. S3 and Supplementary Fig. S4), have been predicted using three algorithms, as described in the methods. *Ap* species possess fewer genes coding ADHs and more genes coding ALDHs than *K. europaeus* 5P3. There are 7, 2, 2, 2, 1, and 1 membrane-bound ADHs, as well as 1, 1, 1, 3, 0 and 0 membrane-bound ALDHs in *K. europaeus* 5P3, *K. oboediens* 174Bp2, *Ap* 386B, *Ap* IFO 3283-32, *Ap* CICC 20001 and *Ap* CGMCC 1.41, respectively. Clearly, compared to the others, *K. europaeus* 5P3 possesses more than 3 times the genes coding membrane-bound ADHs but a similar number of genes coding membrane-bound ALDHs. Such high levels of ADHs, especially PQQ-ADH, may be a key factor that allows *K. europaeus* 5P3 to accumulate such a high concentration of acetic acid. Moreover, during vinegar manufacture, a single gene coding membrane-bound ALDH is able to satisfy demand by producing a high level of acetic acid. Interestingly, *Ap* CICC 20001 and CGMCC 1.41 have one PQQ-ADH, and no membrane-bound ALDH, which may explain why they produce a lower concentration of acetic acid than *K. europaeus* strains. Inserting more copies of genes coding PQQ-ADH to the genome of *Ap* strains may be an effective method to improve their ability to produce a high level of acetic acid.

**Figure 7:**
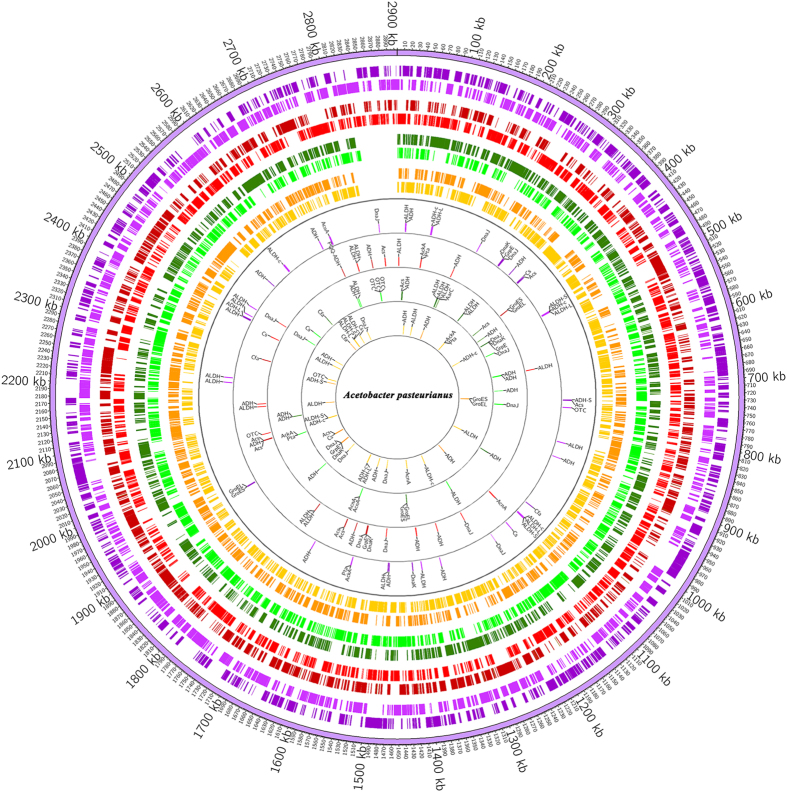
Genes related to producing and tolerating acetic acid in *Ap* IFO 3283-32, 386B, CGMCC 1.41 and CICC 20001. From outer to inner, the first, second, third and fourth circles depict chromosomes of *Ap* IFO 3283-32, CGMCC 1.41, CICC 20001 and 386B. The fifth, sixth, seventh and eighth circles depict genes related to producing and tolerating acetic acid in *Ap* IFO 3283-32, CGMCC 1.41, CICC 20001 and 386B.

The distribution of ADHs and ALDHs are shown in Fig. 7. Genes coding ADHs and ALDHs are distributed throughout the chromosomes of *Ap* strains at regular intervals. Occasionally, ADH and ALDH are clustered as well. This indicates that ADHs and ALDHs are common enzymes for metabolism in *Ap* strains, accumulating acetic acid in the full growth process under specific conditions. Therefore, acid resistance and the expression level of ADHs and ALDHs may be key factors for producing a high concentration of acetic acid.

#### Tactical cooperation may be involved in acetic acid tolerance in Ap strains

The genes coding enzymes that participate in mechanisms conferring acid resistance in microorganisms^15^,^32^,^33^,^34^,^35^ were investigated in *K. europaeus* 5P3, *K. oboediens* 174Bp2, *Ap* 386B, *Ap* IFO 3283-32, *Ap* CICC 20001 and *Ap* CGMCC 1.41 (Supplementary Data S3). As indicated in Table 2, there are more genes coding PQQ-ADHs in *K. europaeus* 5P3 than in *Ap* CICC 20001 and CGMCC 1.41. A high level of PQQ-ADHs, may contribute not only to producing high concentrations of acetic acid but also to tolerating an extreme acid environment. Acetate kinase (AckA), acetyl-CoA synthetase (Acs), citrate synthase (Cs), aconitate hydratase (AcnA) and phosphate acetyltransferase (Pta) participate in acetic acid assimilation in AAB, maintaining a higher intracellular than extracellular pH value. All of these enzymes are involved in the common metabolism in AAB and are similar to those found in *K. europaeus* 5P3, *K. oboediens* 174Bp2, *Ap* 386B, *Ap* IFO 3283-32, *Ap* CICC 20001 and *Ap* CGMCC 1.41. The ADI pathway plays a key role in acid resistance in *Lactobacillus reuteri*^47^. Interestingly, compared to other AAB strains, the ADI pathway is peculiar to *K. europaeus* 5P3. The ADI pathway may be a key factor that allows *K. europaeus* 5P3 to accumulate a high level of acetic acid. Genes coding enzymes that contribute to acetic acid resistance cluster in the chromosome of *Ap* strains. These include molecular chaperones, such as the combination of GroES and GroEL and the combination of DnaK, GrpE and DnaJ, and enzymes concerned with the assimilation of acetic acid, including the combination of Pta and AckA and the combination of Cs and Acs (Fig. 7).

Tactical cooperation may be involved in acetic acid tolerance in *Ap* strains. Because an increasing amount of acetic acid is produced, an extreme acid environment on both sides of the membrane, has been gradually formed. During acetic acid fermentation, a great deal of acetic acid is produced outside and diffuses into the cytoplasm, creating a lower pH environment. To maintain a relatively higher pH value intracellularly, some effective methods are used in *Ap* CGMCC 1.41 cells. First, PQQ-ADH activities are directly related to acetic acid resistance and thermotolerance in AAB^21^,^53^, likely because ADHs act as the main enzyme in energy metabolism of *Ap*. Second, in the cytoplasm, acetic acid is transformed to acetyl-CoA by acetyl-CoA synthetase (AS. 1613–1614, AS. 2105–2106, and AS. 2938), or by acetate kinase (AS. 74) and phosphotransacetylase (AS. 75). Then, acetyl-CoA is transformed to isocitrate by sequential catalytic action of citrate synthase (AS. 2465) and aconitate hydratase (AS. 1043) in the tricarboxylic acid (TCA) cycle. Third, chaperones, such as GroES (AS. 388), GroEL (AS. 389), DnaK (AS. 1527), DnaJ (AS. 1168, AS. 1449, AS. 1528 and AS. 2523) and GrpE (AS. 1525) confer stress resistance to stressors including temperature shifts, ethanol and acetic acid. Fourth, cyclopropane-fatty-acyl-phospholipid synthase (AS. 2381) catalyzes the formation of cyclopropane fatty acid, which is a major component of the phospholipids of many species of gram-negative bacteria and considered a conditional and post-synthetic modification of bacterial membrane lipid bilayers. The formation of cyclopropane fatty acid may lead to a change in the morphology and permeability to acetic acid involved in acetic acid resistance. Fifth, lysine decarboxylase (AS. 1249) and ornithine decarboxylase (AS. 1047) replace the α-carboxyl groups of their amino acid substrates with a proton from the cytoplasm. CO_2_ is produced, as well as either cadaverine, which is the end product of the reaction catalyzed by lysine decarboxylase, or putrescine, the end product of that catalyzed by ornithine decarboxylase. These polyamines, consisting of multi-basic group, provide an alkaline environment in the cytoplasm. Then, the putrescine ABC transporter (AS. 2264–2265, AS. 2726–2728, and AS. 2730) removes the superfluous putrescine. Furthermore, in the amino acid decarboxylation-antiporter system, the cognate antiporters expel the decarboxylation products and import new amino acid substrates.

In *Ap* CICC 20001, similar methods are adopted to maintain a relatively high pH value in the cytoplasm. Acetyl-CoA synthetase (HN. 126, HN. 1654 and HN. 2080), acetate kinase (HN. 787), phosphotransacetylase (HN. 786), citrate synthase (HN. 1277), aconitate hydratase (HN. 2323–2424), GroES (HN. 3179), GroEL (HN. 3196), DnaK (HN. 2159), DnaJ (HN. 1225, HN. 2158, HN. 2234 and HN. 2500), GrpE (HN. 2161), cyclopropane-fatty-acyl-phospholipid (HN. 1360), Ornithine decarboxylase (HN.3419), lysine decarboxylase (HN. 2459) and putrescine ABC transporter (HN. 2264–2265, HN. 2726–2728, HN. 2730) contribute to acetic acid resistance in an extreme acid environment.

#### The metabolic blueprint reveals the approaches to improve acetic acid production and resistance in Ap

**Figure 8:**
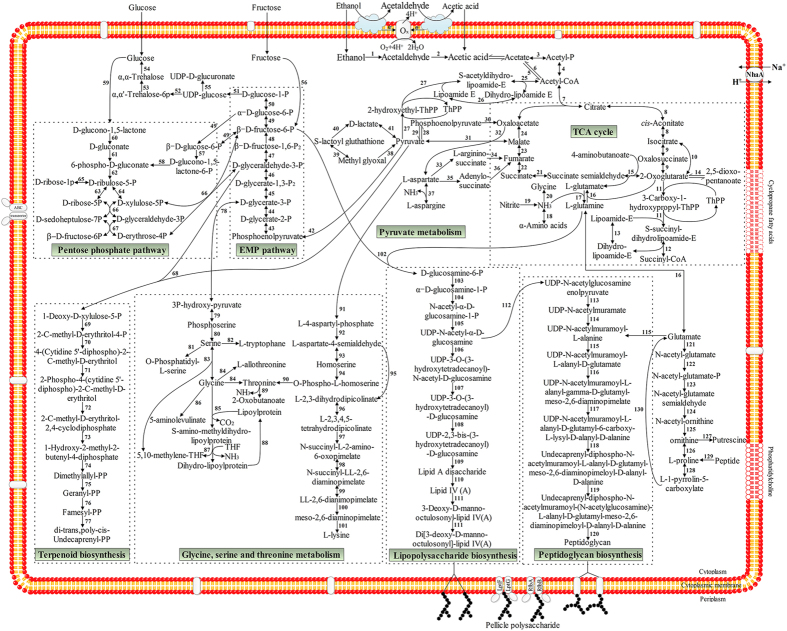
Metabolic pathways related to acetic acid resistance in *Ap* CGMCC 1.41. These include the TCA cycle, pyruvate metabolism, the EMP pathway, the pentose phosphate pathway, terpenoid biosynthesis, glycine metabolism, lipopolysaccharide biosynthesis and peptidoglycan biosynthesis. Enzymes in these pathways are included in Supplementary Text 1.

To understand the overall mechanisms of acetic acid resistance in *Ap*, the main metabolic pathways related to acetic acid tolerance and production in *Ap* CGMCC 1.41 were arranged in combination with the discovered acid-tolerant mechanisms in *E. coli* and the published acetic acid resistance in AAB (Fig. 8). These pathways have been shown to be the common pathways in *Ap* species, including *Ap* CGMCC 1.41 and CICC 20001. In *Ap*, there is a special TCA cycle, in which succinate-semialdehyde dehydrogenase (EC 1.2.1.24), rather than succinyl coenzyme A synthetase (EC 6.2.1.4), is the intermediate enzyme in the transformation of 2-oxoglutarate to succinate. This substitution shortens the process of the TCA cycle, gaining a higher metabolic efficiency. In the metabolic blueprint of *Ap*, pyruvate metabolism is the main process that integrates other metabolic processes, including the special TCA cycle, EMP pathway, pentose phosphate pathway, terpenoid biosynthesis, glycine metabolism and lipopolysaccharide biosynthesis. From these metabolic pathways, *Ap* can use glucose, fructose, ethanol and acetic acid as ideal carbon sources to grow. Although *Ap* species have no uniform mechanisms that confer acid tolerance in *E. coli*, such as the arginine-dependent system, the glutamate-dependent system and the lysine-dependent system, some analogous pathways, as well as published mechanisms, may contribute to acetic acid resistance in *Ap*. In the presence of ammonia-lyases, NH_3_ is produced from ornithine, threonine and S-amino-methyl-dihydrolipoyl protein. Moreover, NH_3_ also can be integrated into L-aspartate and L-glutamate, forming the corresponding amino acids. These opposed reactions may balance the contents of NH_3_ and control the intracellular pH value in cells. Furthermore, ornithine decarboxylase (EC 4.1.1.17) catalyzes the transformation of ornithine to putrescine, which is a polyamine that greatly modifies intramembrane pH in microorganisms^54^.

### The genetic stabilities of *Ap* strains may vary with the individual

Similar to other organisms, the genome of *Ap* strains is remarkably stable from one generation to the next but is plastic on an evolutionary timescale. Bacterial chromosomes are complex and dynamic, thereby maintaining a balance between genome integrity and instability and allowing the survival of organisms and their offspring^55^. Genomic rearrangements, including deletions, duplications, amplifications, insertions, inversions and translocations, lead to instability of the genome. Some of these mutations are silent, while others bring about phenotypic variation, evolution and speciation. Genomic instability plays two roles in organismal survival. On the one hand, specialized genetic elements, including mobile elements, inteins, introns, retroelements and integrons, and recombination methods, homologous or illegitimate, can mediate genome instability, generating phenotypic variation; on the other hand, restriction-modification (RM) systems and the CRISPR-Cas system (comprising CRISPR and CRISPR-associated proteins) use genome instability to protect organisms from invasion by phages and mobile elements^56^,^57^. Mobile elements, widely present in organisms, contain insertion sequences (IS), miniature inverted-repeat transposable elements (MITEs), repetitive extragenic palindromic (REP) sequences, bacterial interspersed mosaic elements (BIMEs), transposable elements (TEs), transposable bacteriophages and genomic islands and involve genome instability. The long terminal repeats (LTRs) of *Ap* were predicted using LTR Finder (Supplementary Data S4). *Ap* CGMCC 1.41 and all *Ap* 3283 substrains have an LTR of 1269 bp in the chromosome and 833 bp in the plasmid, respectively. Meanwhile, *Ap* 386B and *Ap* CICC 20001 have no LTR in their genomes. Like LTR, all *Ap* 3283 substrains have a confirmed CRISPR element (Supplementary Data S5) of 1431 bp in almost the same location of the genome, and *Ap* 386B has no CRISPR element in either its chromosome or its plasmids. The chromosomes of *Ap* CGMCC 1.41 and *Ap* CICC 20001, have 1 and 2 putative CRISPR elements, respectively. Furthermore, *Ap* CICC 20001 have a large plasmid containing two confirmed CRISPR elements and four putative CRISPR elements. This plasmid may greatly contribute to the gene stability of *Ap* CICC 20001 by escaping insertions from phages and mobile elements.

Stability and instability factors direct the balance between genome integrity and instability. As previously mentioned, after 30 years of storage, 7 substrains with different phenotypic characteristics were isolated from *Ap* IFO 3283. Moreover, when one of these substrains IFO 3283-01 was exposed to a high temperature, a mutant strain IFO 3283-01-42C, which tolerated temperatures as high as 42 °C, was isolated and sequenced. Compared to other substrains, a DNA fragment is missing in *Ap* 3283-01-42C. Further genomic analysis showed that the genomes of all *Ap* IFO 3283 substrains contained more than 280 transposons and five genes with hyper-mutable tandem repeats, revealing the genetic instability of *Ap*^26^. However, Gullo, *et al.*^27^ believed that *Ap* strains are stable because at different ages of the culture and frequencies of subculture, *Ap* AB0220 showed a high stability over 9 years of preservation. Instability and stability factors in the genome of *Ap* strains were summarized (Table 3).

There are approximately 270 transposases and 1 LTR in the genomes of *Ap* IFO 3283 substrains, while *Ap* 386B, *Ap* CICC 20001 and *Ap* CGMCC 1.41 possess fewer transposases or mobile elements than do the *Ap* IFO 3283-32 substrains. Importantly, *Ap* CICC 2001 contains 5 CRISPR elements that contribute to genome integrity and no more than 80 transposases that relate to genetic instability. Therefore, *Ap* CICC 20001 may have a very stable genome. The stability of *Ap* strains may vary with the individual. In a given environment, the balance of stability factors and instability factors control the stability of *Ap* strains.

## Discussion

*Ap* CICC 20001 and CGMCC 1.41 display strong abilities in both producing and tolerating acetic acid, with more than 6%, by liquid-state fermentation. Furthermore, *Ap* CGMCC 1.41 tolerates higher concentrations of ethanol and acetic acid, and produces a high level of acetic acid in the modified GY medium in an Erlenmeyer flask. However, the ability to produce acetic acid in *Ap* strains may vary by each individual strain. *Ap* CICIM B7003, which was isolated from industrial vinegar bioreactors in China, yielded 7.00% final acetic acid in a semi-continuous regime by an optimized protocol, which was less than *Ap* CGMCC 1.41^22^. However, *Ap* CICIM B7003-02, an ultraviolet mutant from *Ap* CICIM B7003, produces a high acidity vinegar with an acetic acid concentration that reached up to 9.33% in the semi-continuous mode in the Frings Pilot-Acetator 9 L^58^. A bioreactor is a more effective piece of equipment for brewing vinegar than an Erlenmeyer flask. This study indicates that *Ap* CGMCC 1.41 may be an ideal strain for producing high levels of acetic acid (up to 9%).

Comparative genomic analysis of 11 *Ap* strains reveals that the chromosomes of *Ap* CICC 20001 and CGMCC 1.41 are evolutionarily conserved, sharing a high degree of homology with other *Ap* strains, whereas their plasmids are unique, suggesting a separate evolution between *Ap* chromosomes and plasmids. All of the *Ap* strains also share almost identical proportions of amino acid components in their genomes. All IFO 3283 substrains possess an almost identical chromosome, although IFO 3283-01-42C lost a DNA fragment and tolerates a higher temperature. In these substrains, 4 transposon insertions (SecB2, glycosyl transferase, two component kinase and intergenic), 3 SNPs (glycerol kinase, RopA and hypothetical) and 3 hyper-mutable Tandem Repeats (HTRs) were identified as chromosomal variations, while a transposon insertion and a HTR were observed in the largest plasmid^26^.

Our results comparing the numbers of PQQ-ADH in 3 AABs with a range of abilities to produce acetic acid emphasize that PQQ-ADH contributes to acetic acid tolerance in *Ap*. Comparison of genes related to acetic acid resistance in AAB reveals that acetate kinase, acetyl-CoA synthetase, citrate synthase, aconitate hydratase and phosphate acetyltransferase jointly participate in acetic acid assimilation in *Ap*, resisting acetic acid in the presence of a high concentration of acetic acid. Furthermore, the pathway related to acetic acid tolerance shows that in addition to reported mechanisms conferring acetic acid resistance, metabolism of some amino acids, such as degradation of threonine, glycine and ornithine, contribute to acetic acid tolerance by producing a large amount of NH_3_, which decreases the intracellular pH value, as in *E. coli*. Ornithine also can be degraded and transformed to putrescine, greatly neutralizing intracellular pH in microorganisms^54^. Moreover, 3 urease genes were detected in *Ap*, but no urease gene was found in *K. europaeus* 5P3 and *K. oboediens* 174Bp2 (Supplementary Data S3), which indicates that urease system may additionally contribute to acetic acid resistance of *Ap*. The blueprint constructed in this study benefits the investigation of acetic acid resistance and related regulation.

All *Ap* strains contain some transposases or mobile elements causing genetic instability and several protection systems, including CRISPR and RM involved in genetic stability by avoiding insertions from phages and mobile elements. A balance between protection systems and transposases directs genetic stability and instability. *Ap* CICC 20001 and CGMCC 1.41 have been utilized to steadily brew vinegar in the vinegar industry in China for more than 50 years. *Ap* CICC 20001 and CGMCC1.41 may be more stable than IFO 3283 substrains because genomes of *Ap* CICC 20001 and CGMCC1.41 contain one-third of the transposases and a greater number of protective systems than the genome of IFO 3283 substrains. The genetic stability of *Ap* strains may vary by individual.

In summary, we uncovered global insights into acetic acid resistance mechanisms and genetic stability of *Ap* strains using comparative genomics. These observations provide important insights into the evolution, acid resistant mechanism, and genetic stability of these two economically important AAB strains and lay a foundation for future genetic manipulation and engineering of these strains. However, the acid-tolerant mechanisms have only been predicted by comparing the genes related to acid resistance in other microorganisms, without experimental verification. Verifying the mechanisms conferring acetic acid resistance and related regulation systems should be hot topics in the study of AAB.

## Methods

### Strains and growth conditions

The AAB strains used in this study are *Ap* CICC 20001 and CGMCC1.41 from the China Center of Industrial Culture Collection (CICC) and China General Microbiological Culture Collection Center (CGMCC), respectively. *Ap* CICC 20001 and CGMCC 1.41 were grown in glucose yeast medium (10% glucose and 1% yeast extract) and bean sprout glucose ethanol medium (20% bean sprout extract, 1% glucose and 2% ethanol) and then cultivated on an incubator shaker for 24 h at 30 °C. The cells were harvested by centrifugation at 9000 × g for 10 min. *Ap* CICC 20001 and CGMCC 1.41 were inoculated on GYC medium (5% glucose, 1% yeast extract and 2% CaCO_3_) using the streak method.

### Fermentation characteristics of *Ap* CICC 20001 and CGMCC 1.41 strains

*Ap* CICC 20001 and CGMCC1.41 were grown in a modified GYP medium (0.1% glucose, 0.2% peptone, 0.5% yeast extract) containing different concentrations of ethanol (0, 2%, 4%, 6%, 8%, 10% and 12%) or different concentrations of acetic acid (0, 0.5%, 1%, 1.5%, 2%, 2.5%, 3%, 3.5% and 4%). An inoculum of 250 μl (approximately A_600_ = 0.5) was inoculated in a 250 ml Erlenmeyer flask containing 50 ml GYP medium with 3% ethanol. When the exponential growth phase was reached, 5 ml of this culture was used as an inoculum for acetic acid fermentation in a 250 ml Erlenmeyer flask containing 50 ml GYP medium with a defined concentration of acetic acid and ethanol. The inoculated flasks were incubated on the rotary shaker at 170 rpm and 30 °C. The acidity of the medium was measured with 0.1 M NaOH using phenolphthalein as an indicator.

### Genomic sequencing

*Ap* CICC 20001 and CGMCC 1.41 cells were subjected to SMRT sequencing at the Institute of Medicinal Plant Development (Beijing, China) using a PacBio RS DNA sequencer (Pacific Biosciences, Menlo Park, CA, USA; http://www. pacificbiosciences.com/)^59^. SMRT bell template libraries with DNA fragments of 2 kb were prepared^60^. Then, sequencing was performed by utilizing one SMRT cell (http://www.pacificbiosciences.com/products/consumables/SMRT-cells/), and obtaining a zero-mode waveguide^61^. SMRT reads were mapped to the *Ap* genome reference sequence using BLASR software (http://github.com/PacificBiosciences/blasr)^62^ according to standard mapping protocols. Interpulse durations were measured as described for all of the pulses aligned to each position in the *Ap* genome sequence. The modified bases were identified applying the SMRT Analysis Server v.1.4.0 (Pacific Biosciences). Genomic sequences were assembled using SMRT analysis RS_HGAP_Assembly.2 (https://github.com/PacificBiosciences/SMRT-Analysis/wiki/SMRT-Pipe-Reference-Guide-v2.2.0#PRO_HGAP2). Automatic gene prediction and annotation of the assembled genome sequences were performed using RAST (http://rast.nmpdr.org/)^63^. The annotated genes were classified using the Clusters of Orthologous Groups of proteins (COG) database (http://www.ncbi.nlm.nih.gov/COG/). The pathways that genes participate in were analyzed using the Kyoto Encyclopedia of Genes and Genomes (KEGG) database (http://www.genome. jp/kegg/). The generated data are available for download at the website http://fbfs.hzau.edu.cn/AAB/mulu/genome.asp.

### Visualization of data

CGView (http://stothard.afns.ualberta.ca/cgview_server/)^64^ was utilized to exhibit graphical layouts of the chromosome, as well as the corresponding GC content and GC skew. Circos 0.66 (http://circos.ca/) was used to highlight the distribution of the genes contributing to the production and tolerance of acetic acid in *Ap* genomes. Metabolic pathways in *Ap* CGMCC 1.41 were mapped using the KEGG PATHWAY database and expressed using Edrawmax-cn_7.2.

### Prediction of special genes or structures

Essential genes were predicted using ZCURVE 3.0 software^65^ (http://cefg.uestc.edu.cn/zcurve/). To identify membrane-bound ADH and ALDH, Kyte-doolittle (http://gcat.davidson.edu/DGPB/kd/kyte-doolittle.htm), Tmpred (http://www.ch.embnet.org/software/TMPRED_form.html) and TMHMM 2.0 (http://www.cbs.dtu.dk/services/TMHMM/), which are based on hydrophobicity, a transmembrane proteins database and Hidden Markov Models, respectively, were utilized to predict the transmembrane helices of ADHs and ALDHs of *Ap* CICC 20001, *Ap* CGMCC 1.41, *Ap* IFO 3283-32, *Ap* 386B, *K. europaeus* 5P3 and *K. oboediens* 174Bp2. Tandem Repeats Finder software (http://tandem.bu.edu/trf/trf.basic.submit.html), LTR Finder software (http://tlife.fudan.edu.cn/ltr_finder/index.php) and CRISPR finder software (http://crispr.u-psud.fr/Server) were applied to predict tandem repeats (TRs), long terminal repeat (LTR) retrotransposons and clustered regularly interspaced short palindromic repeats (CRISPR) in genomes of *Ap* strains, respectively.

### Orthology analysis

The previously published genome sequences of *Ap* IFO 3283-01, IFO 3283-01-42C, IFO 3283-03, IFO 3283-07, IFO 3283-12, IFO 3283-22, IFO 3283-26 and IFO 3283-32 were downloaded from NCBI. Mauve 2.3.1 (http://darlinglab.org/mauve/mauve.html) was used to compare genome orthologies of *Ap* CICC 20001 and CGMCC 1.41 with these *Ap* species. To compare orthologous plasmids, plasmids in all of these *Ap* species were integrated severally.

## Additional Information

**How to cite this article**: Wang, B. *et al.* Global insights into acetic acid resistance mechanisms and genetic stability of *Acetobacter pasteurianus* strains by comparative genomics. *Sci. Rep.* **5**, 18330; doi: 10.1038/srep18330 (2015).

## Supplementary Material

Supplementary Information

## Figures and Tables

**Table 1:** Genomic features of *Ap* CICC 20001 and CGMCC 1.41.

	*Ap*CICC 20001	*Ap* CGMCC 1.41
Chromosome		
Size (bp)	2865612	2928931
G + C%	52.94%	52.95%
CDSs	3006	3012
Essential genes	234	146
tRNA	118	111
rRNA	34	30
Prophages	2	2
Mobile elements	15	56
Plasmids		
Number	10	7
CDSs	617	237
G + C%	47.78	52.53
tRNA	20	0
rRNA	6	0
Mobile elements	59	44
Total hypothetical genes	1173	1002
Total genome CDSs	3623	3250

**Table 2:** Gene numbers of membrane-bound ADHs and ALDHs in different AAB strains.

Strains	PQQ-ADH /Total ADH	PQQ-ALDH /Total ALDH
*K. europaeus* 5P3	7/20	1/7
*K. oboediens* 174Bp2	2/14	1/5
*A. pasteurianus* 386B	2/8	1/7
*A. pasteurianus* IFO 3283-32	2/13	3/14
*A. pasteurianus* CICC 20001	1/10	0/9
*A. pasteurianus* CGMCC 1.41	1/8	0/9

**Table 3:** Factors related to instability and stability in chromosome (Chr) and plasmid (Plsm) of *Ap* strains.

*Ap* strains	CRISPER	RM	LTR	Transposases	Mobile elements
3283-01	1(Plsm)	11	1	270	0
3283-01-42C	1(Plsm)	10	1	254	0
3283-03	1(Plsm)	11	1	269	0
3283-07	1(Plsm)	11	1	268	0
3283-12	1(Plsm)	11	1	267	0
3283-22	1(Plsm)	11	1	269	0
3283-26	1(Plsm)	11	1	269	0
3283-32	1(Plsm)	11	1	267	0
386B	0	6	0	49	0
CICC 20001	2(Chr) + 3(Plsm)	2	0	5	74
CGMCC 1.41	1(Chr)	9	1	4	100
