# Supplementary material for: Global insights into acetic acid resistance mechanisms and genetic stability of Acetobacter pasteurianus strains by comparative genomics
Source: Sci Rep. 2015 Dec 22;5:18330. doi: 10.1038/srep18330 (PMC4686929; doi:10.1038/srep18330)
Supplement: Supplementary Information [file srep18330-s1.doc]

**Supplementary Information for**

**Global insights into acetic acid resistance mechanisms and genetic stability of *Acetobacter pasteurianus* strains by comparative genomics**

Bin Wang^3^, Yanchun Shao^3^, Tao Chen^3^, Wanping Chen^1,3*^, Fusheng Chen^1,2,3*^

1. Key Laboratory of Environment Correlative Dietology, Huazhong Agricultural University, Wuhan 430070, Hubei Province, P. R. China;

2. Hubei Provincial Cooperative Innovation Center of Industrial Fermentation, Hubei University of Technology, Wuhan 430068, Hubei Province, P. R. China;

3. College of Food Science and Technology, Huazhong Agricultural University, Wuhan 430070, Hubei Province, P. R. China

* Corresponding authors

E-mail: WC, chenwanping@mail.hzau.edu.cn; FC, chenfs@mail.hzau.edu.cn

Tel.: 86-27-87282111

Contents

[Supplementary Text 3](#__RefHeading___Toc428194931)

[Text S1 | Main enzymes in metabolic pathway related to acetic acid resistance in *Ap* CGMCC 1.41. 3](#__RefHeading___Toc428194932)

[Supplementary Figures 6](#__RefHeading___Toc428194933)

[Figure S1 | Clusters of orthologous groups in *Ap* CICC 20001 and CGMCC 1.41. 6](#__RefHeading___Toc428194934)

[Figure S2 | Essential genes in *Ap* IFO 3283-32, 386B, CGMCC 1.41 and CICC 20001 7](#__RefHeading___Toc428194935)

[Figure S3 | The predicted topologies of ADHs in the AABs. 9](#__RefHeading___Toc428194936)

[Figure S4 | The predicted topologies of ALDHs in the AABs. 10](#__RefHeading___Toc428194937)

[Supplementary Data 11](#__RefHeading___Toc428194938)

[Data S1 | The number of ADHs predicted by three methods in AABs. 11](#__RefHeading___Toc428194939)

[Data S2 | The number of ALDHs predicted by three methods in AABs. 14](#__RefHeading___Toc428194940)

[Data S3 | The number of genes related to acetic acid resistance in AABs. 16](#__RefHeading___Toc428194941)

[Data S4 | Prediction of LTR in *Ap* species. 17](#__RefHeading___Toc428194942)

[Data S5 | Prediction of CRISPER in *Ap* species. 18](#__RefHeading___Toc428194943)

# Supplementary Text

## Text S1 | Main enzymes in metabolic pathway related to acetic acid resistance in *Ap* CGMCC 1.41.

(1) Alcohol dehydrogenase (AS. 170, 1279, 1568, 2227, 2801 and 2899, EC 1.1.1.1); (2) Aldehyde dehydrogenase (AS. 5, 603, 1575, 2219, 2849, 2850, 3011 and 3012, EC 1.2.1.3); (3) Acetate kinase (AS. 74, EC 2.7.2.1); (4) Phosphate acetyltransferase (AS. 75, EC 2.3.1.8); (5) Acetyl-CoA synthetase (AS. 1613, 1614, 2105, 2106 and 2938, EC 6.2.1.1); (6) Acetyl-CoA hydrolase (AS. 2470, EC 3.1.2.1); (7) Citrate synthase (AS. 2465, EC 2.3.3.1); (8) Aconitate hydratase (AS. 1043, EC 4.2.1.3); (9) Isocitrate dehydrogenase [NADP] (AS. 2005, EC1.1.1.42); (10) Isocitrate dehydrogenase [NAD] (AS. 2437, EC 1.1.1.41); (11) 2-Oxoglutarate dehydrogenase E1 component (AS. 2163, EC 1.2.4.2); (12) Dihydrolipoamide succinyltransferase component (E2) of 2-oxoglutarate dehydrogenase complex (AS. 2164, EC 2.3.1.61); (13) Dihydrolipoamide dehydrogenase of 2-oxoglutarate dehydrogenase (AS. 2165, EC 1.8.1.4); (14) 2-Ketoglutaric semialdehyde dehydrogenase (AS. 796, EC 1.2.1.26); (15) Gamma-aminobutyrate: alpha-ketoglutarate aminotransferase (AS. 1065, EC 2.6.1.19); (16) Glutamate synthase [NADPH] large chain (AS. 573 and 574, EC 1.4.1.13); (17) Glutamine synthetase type I (AS. 2011, EC 6.3.1.2); (18) D-amino acid dehydrogenase small subunit (AS. 1025 and 2001, EC 1.4.99.1); (19) Nitrite reductase [NAD(P)H] large subunit (AS. 2855, EC 1.7.1.4); (20) Urea carboxylase-related aminomethyltransferase (AS. 2808 and 2809, EC 2.1.2.10); (21) Succinate- semialdehyde dehydrogenase [NAD] (AS. 615, 1278, 2743, 2900 and 3035, EC 1.2.1.24); (22) Succinate dehydrogenase (AS. 1285-1288, EC 1.3.99.1); (23) Fumarate hydratase (AS. 56, 58, and 1022, EC 4.2.1.2); (24) Malate:quinone oxidoreductase (AS. 2566, EC 1.1.5.4); (25) Dihydrolipoamide acetyltransferase component of pyruvate dehydrogenase complex (AS. 2630, EC 2.3.1.12); (26) Dihydrolipoamide dehydrogenase of 2-oxoglutarate dehydrogenase (AS. 2165 and 2629, EC 1.8.1.4); (27) Pyruvate dehydrogenase E1 component (AS. 2631 and 2632, EC 1.2.4.1); (28) Pyruvate phosphate dikinase (AS. 487, EC 2.7.9.1); (29) Pyruvate kinase (AS. 1993, EC 2.7.1.40); (30) Phosphoenolpyruvate carboxylase (AS. 265, EC 4.1.1.31); (31) NAD-dependent malic enzyme (AS. 1374 and 1375, EC 1.1.1.38); (32) Aspartate aminotransferase (AS. 538, EC 2.6.1.1); (33) Arginino-succinate synthase (AS. 102, EC 6.3.4.5); (34) Argininosuccinate lyase (AS. 2234, EC 4.3.2.1); (35) Adenylosuccinate synthetase (AS. 1557-1558, EC 6.3.4.4); (36) Adenylosuccinate lyase (AS. 2621, EC 4.3.2.2); (37) L-asparaginase (AS. 155, EC 3.5.1.1) or Asparagine synthetase (AS. 342, EC 6.3.5.4); (38) Aldehyde dehydrogenase A (AS. 1736 and 1737, EC 1.2.1.22); (39) Lactoylglutathione lyase (AS. 2047, EC 4.4.1.5); (40) Hydroxyacylglutathione hydrolase (AS. 2172, EC 3.1.2.6); (41) D-lactate dehydrogenase (AS. 604, EC 1.1.2.5 and AS. 751, EC 1.1.2.4); (42) Pyruvate kinase (AS. 1993, EC 2.7.1.40); (43) Enolase (AS. 2505 and 2506, EC 4.2.1.11); (44) Fructose-2,6-bisphosphatase (AS. 2201, EC 5.4.2.1); (45) Phosphoglycerate kinase (AS. 1292, EC 2.7.2.3); (46) NAD-dependent glyceraldehyde-3-phosphate dehydrogenase (AS. 1293, EC 1.2.1.12); (47) Fructose-bisphosphate aldolase class I (AS. 1504, EC 4.1.2.13); (48) Fructose-1,6-bisphosphatase GlpX type (AS. 691, EC 3.1.3.11); (49) Glucose-6-phosphate isomerase (AS. 860, EC 5.3.1.9); (50) Phosphoglucomutase (AS. 189, EC 5.4.2.2); (51) UTP-glucose-1-phosphate uridylyltransferase (AS. 2527, EC 2.7.7.9); (52) Alpha-trehalose-phosphate synthase [UDP-forming] (AS. 1471, EC 2.4.1.15); (53) Trehalose-6-phosphate phosphatase (AS. 1470, EC 3.1.3.12); (54) Trehalase (AS. 2690, EC 3.2.1.28)；(55) UDP-glucose dehydrogenase (AS. 400, EC 1.1.1.22); (56) Fructokinase (AS. 1388, EC 2.7.1.4); (57) Glucose-6-phosphate 1-dehydrogenase (AS. 462, EC 1.1.1.49); (58) 6-phospho- gluconolactonase (AS. 863, EC 3.1.1.31); (59) Glucose dehydrogenase PQQ-dependent (AS. 62, EC 1.1.5.2); (60) Gluconolactonase (AS. 1995, EC 3.1.1.17); (61) Gluconokinase (AS. 865, EC 2.7.1.12); (62) 6-phosphogluconate dehydrogenase decarboxylating (AS. 861, EC 1.1.1.44); (63) Ribose 5-phosphate isomerase A (AS. 864, EC 5.3.1.6); (64) Ribulose-phosphate 3-epimerase (AS. 2245, EC 5.1.3.1); (65) Phosphoglucomutase (AS. 189, EC 5.4.2.2); (66) Transketolase (AS. 858 and 859, EC 2.2.1.1); (67) Transaldolase (AS. 860, EC 2.2.1.2); (68) 1-deoxy-D-xylulose 5-phosphate synthase (AS. 98 and 1653, EC 2.2.1.7); (69) 1-deoxy-D-xylulose-5- phosphate reductoisomerase (AS. 2038, EC 1.1.1.267); (70) 2-C-methyl-D-erythritol 4-phosphate cytidylyltransferase (AS. 361, EC 2.7.7.60); (71) 4-diphosphocytidyl -2-C-methyl-D-erythritol kinase (AS. 1664 and 1665, EC 2.7.1.148); (72) 2-C-methyl-D-erythritol-2,4-cyclodiphosphate synthase (AS. 362, EC 4.6.1.12); (73) 1-hydroxy-2-methyl-2-(E)-butenyl 4-diphosphate synthase (AS. 2540, EC 1.17.7.1); (74) 4-hydroxy-3-methylbut-2-enyl diphosphate reductase (AS. 566, EC 1.17.1.2); (75) Dimethylallyltransferase (AS. 2460, EC 2.5.1.1); (76) (2E,6E)-farnesyl diphosphate synthase (AS. 97, EC 2.5.1.10); (77) Undecaprenyl diphosphate synthase (AS. 2036, EC 2.5.1.31); (78) D-3-phosphoglycerate dehydrogenase (AS. 2605, EC 1.1.1.95); (79) Phosphoserine aminotransferase (AS. 1276 and 1277, EC 2.6.1.52); (80) Phosphoserine phosphatase (AS. 500, EC 3.1.3.3); (81) CDP-diacylglycerol- serine O-phosphatidyltransferase (AS. 2180, EC 2.7.8.8); (82) Tryptophan synthase beta chain (AS. 1552 and 1553, EC 4.2.1.20); (83) Serine hydroxymethyltransferase (AS. 518, EC 2.1.2.1); (84) Low-specificity L-threonine aldolase (AS. 1622, EC 4.1.2.5); (85) Glycine dehydrogenase [decarboxylating] (glycine cleavage system P protein) (AS. 946, EC 1.4.4.2); (86) 5-aminolevulinate synthase (AS. 1296, EC 2.3.1.37); (87) Aminomethyltransferase (glycine cleavage system T protein) (AS. 948, EC 2.1.2.10); (88) Dihydrolipoamide dehydrogenase of pyruvate dehydrogenase complex (AS. 2629, EC 1.8.1.4); (89) Threonine dehydratase (AS. 2366, EC 4.3.1.19); (90) Threonine synthase (AS. 280, EC 4.2.3.1); (91) Aspartokinase (AS. 2537, EC 2.7.2.4); (92) Aspartate-semialdehyde dehydrogenase (AS. 1284, EC 1.2.1.11); (93) Homoserine dehydrogenase (AS. 690, EC 1.1.1.3); (94) Homoserine kinase (AS. 565, EC 2.7.1.39); (95) 4-hydroxy-tetrahydrodipicolinate synthase (AS. 2599 and 2600, EC 4.3.3.7); (96) 4-hydroxy-tetrahydrodipicolinate reductase (AS. 1529, EC 1.17.1.8); (97) 2,3,4,5-tetrahydropyridine-2,6-dicarboxylate N-succinyl-transferase (AS. 2055, EC 2.3.1.117); (98) Acetylornithine aminotransferase (AS. 2129, EC 2.6.1.11); (99) N-succinyl-L,L-diaminopimelate desuccinylase (AS. 2056, EC 3.5.1.18); (100) Diaminopimelate epimerase (AS. 993, EC 5.1.1.7); (101) Diaminopimelate decarboxylase (AS. 2232, EC 4.1.1.20); (102) Glucosamine-fructose-6-phosphate aminotransferase [isomerizing] (AS. 221, EC 2.6.1.16); (103) Phosphoglucosamine mutase (AS. 1141, EC 5.4.2.10); (104) Glucosamine-1-phosphate N-acetyltransferase (AS. 220, EC 2.3.1.157); (105) N-acetylglucosamine-1-phosphate uridyl-transferase (AS. 220, EC 2.7.7.23); (106) Acyl-[acyl-carrier- protein]-UDP-N-acetylglucosamine O-acyltransferase (AS. 2044, EC 2.3.1.129); (107) UDP-3-O-[3-hydroxymyristoyl] N-acetylglucosamine deacetylase (AS. 482, EC 3.5.1.108); (108) UDP-3-O- [3-hydroxymyristoyl] glucosamine N-acyltransferase (AS. 2042, EC 2.3.1.191); (109) Lipid-A-disaccharide synthase (AS. 345, EC 2.4.1.182); (110) Tetraacyldisaccharide 4'-kinase (AS. 2579, EC 2.7.1.130); (111) 3-deoxy-D-manno-octulosonic- acid transferase (AS. 2578, EC 2.-.-.-); (112) UDP-N-acetylglucosamine 1-carboxyvinyltransferase (AS. 1643, EC 2.5.1.7); (113) UDP-N-acetylenolpyruvoyl -glucosamine reductase (AS. 476, EC 1.1.1.158); (114) UDP-N-acetylmuramate- alanine ligase (AS. 475, EC 6.3.2.8); (115) UDP-N-acet ylmuramoylalanine- D-glutamate ligase (AS. 472, EC 6.3.2.9); (116) UDP-N- acetylmuramoylalanyl- D-glutamate--2, 6-diaminopimelate ligase (AS. 469, EC 6.3.2.13); (117) UDP-N- acetylmuramoylalanyl-D-glutamyl-2,6-diaminopimelate- D-alanyl-D-alanine ligase (AS. 470, EC 6.3.2.10); (118) Phospho-N-acetylmuramoyl- pentapeptide-transferase (AS. 471, EC 2.7.8.13); (119) UDP-N-acetylglucosamine- N-acetylmuramyl- S(pentapeptide) pyrophosphoryl- undecaprenol N-acetylglucosamine transferase (AS. 474, EC 2.4.1.227); (120) D-alanyl-D-alanine carboxypeptidase (AS. 1514, EC 3.4.16.4); (121) N-acetylglutamate synthase (AS. 2584, EC 2.3.1.1); (122) Acetylglutamate kinase (AS. 2054, EC 2.7.2.8); (123) N-acetyl-gamma-glutamyl- phosphate reductase (AS. 1575, EC 1.2.1.38); (124) Acetylornithine aminotransferase (AS. 2129, EC 2.6.1.11); (125) Acetylornithine deacetylase (AS. 2845, EC 3.5.1.16); (126) Ornithine cyclodeaminase (AS. 2218, EC 4.3.1.12); (127) Ornithine decarboxylase (AS. 1047, EC 4.1.1.17); (128) Pyrroline-5-carboxylate reductase (AS. 1172, EC 1.5.1.2); (129) Proline iminopeptidase (AS. 410 and 411, EC 3.4.11.5); (130) Delta-1-pyrroline-5- carboxylate dehydrogenase (AS. 2101, EC 1.5.1.12).

# Supplementary Figures


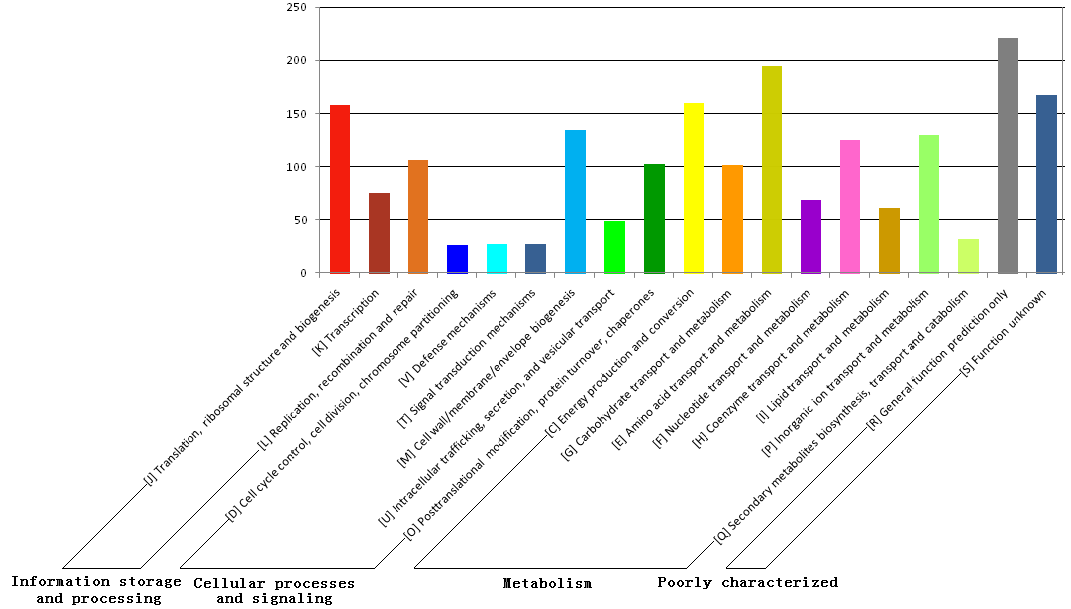
 A


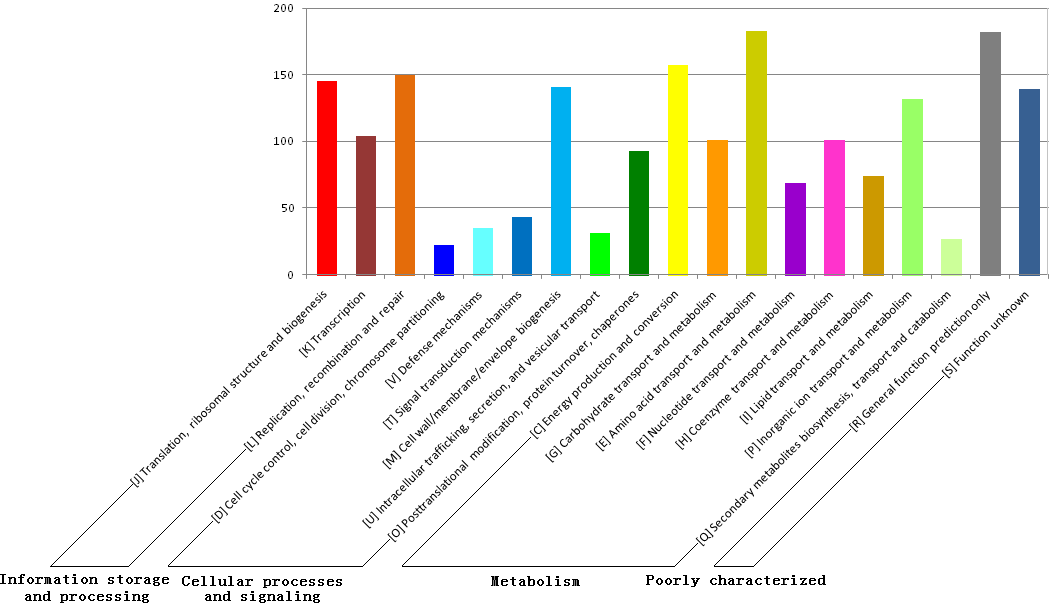


B

## Figure S1 | Clusters of orthologous groups in *Ap* CICC 20001 and CGMCC 1.41.

A and B represent the clusters of orthologous groups in *Ap* CICC 20001 and CGMCC 1.41, respectively.


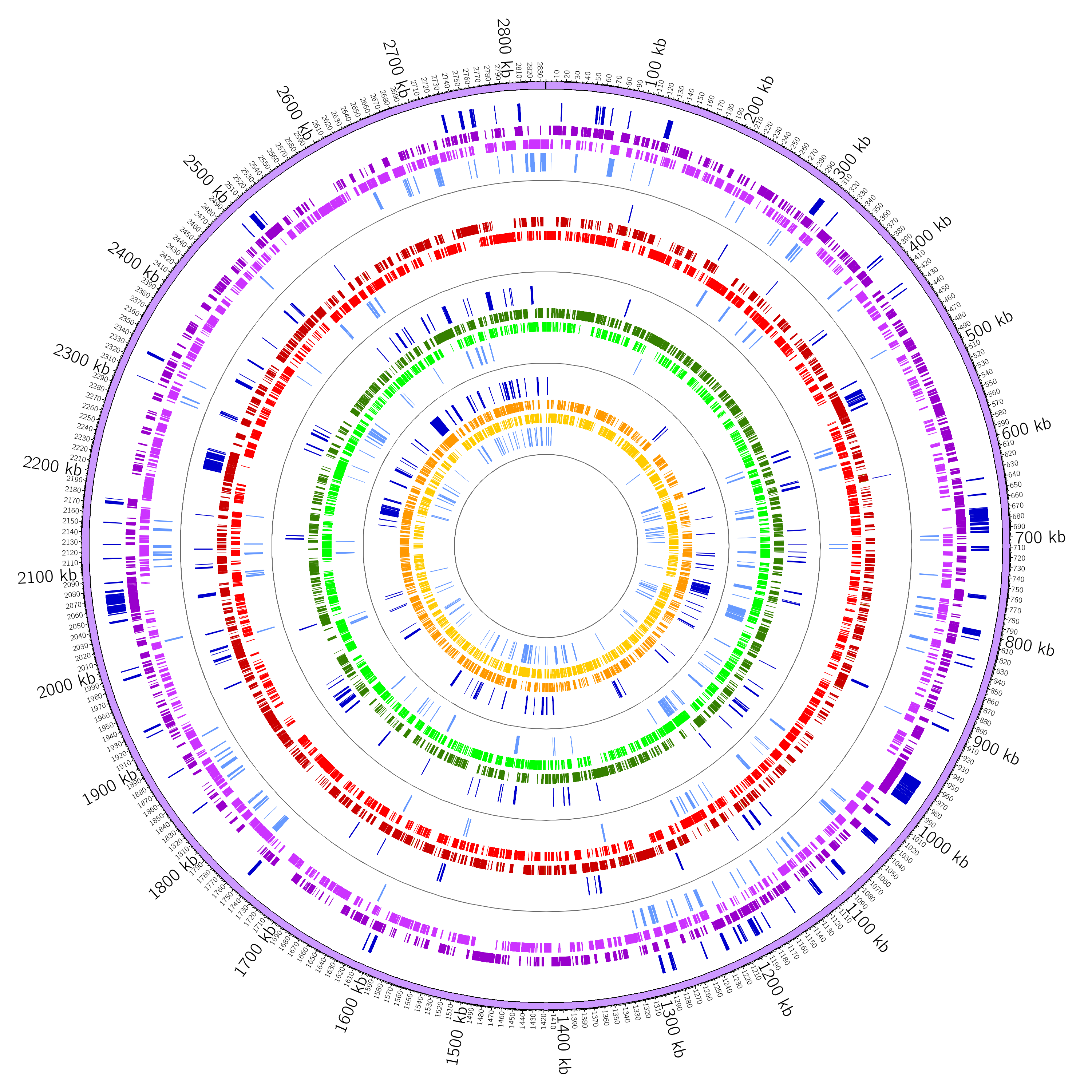


Figure S2 | Essential genes in *Ap* IFO 3283-32, 386B, CGMCC 1.41 and CICC 20001. Chromosomes and essential genes of *Ap* IFO 3283-32, CGMCC 1.41, CICC 20001 and 386B are separated into four hybrid rings from outer to inner by four solid black lines. In each hybrid ring, from outer to inner circle, the first, second, third and fourth circles depict the essential genes on the positive strand, the positive strand of chromosomes, the negative strand of chromosomes and the essential genes on negative strand.


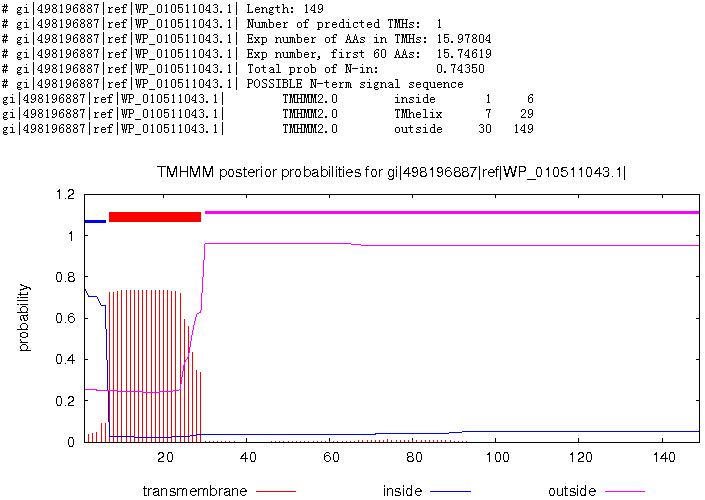

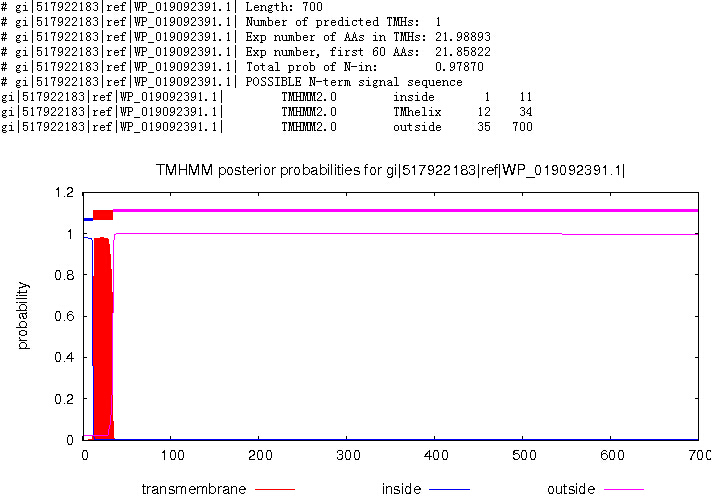
A B


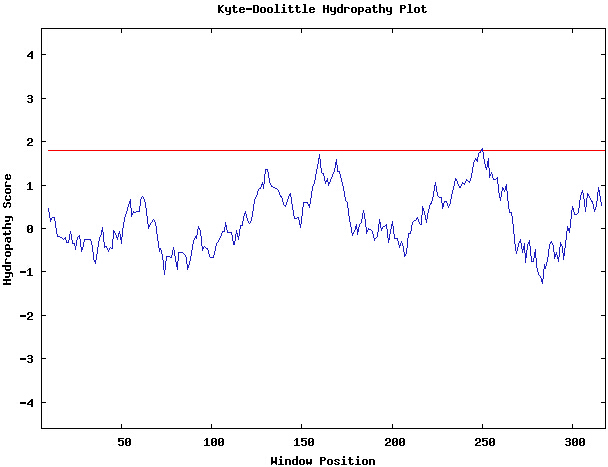

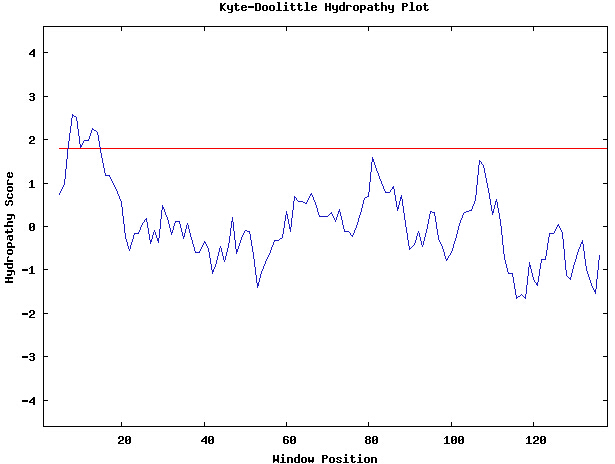
C D


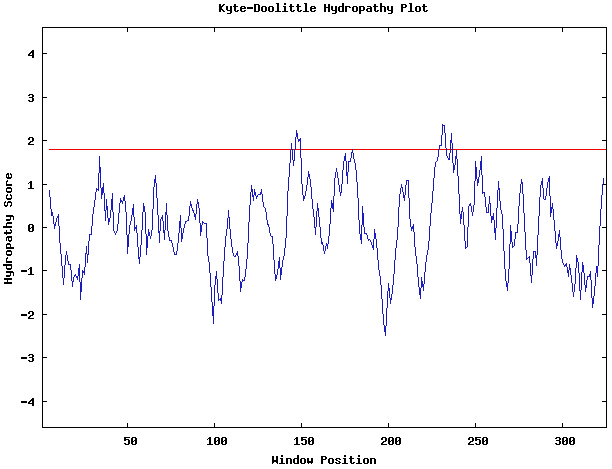

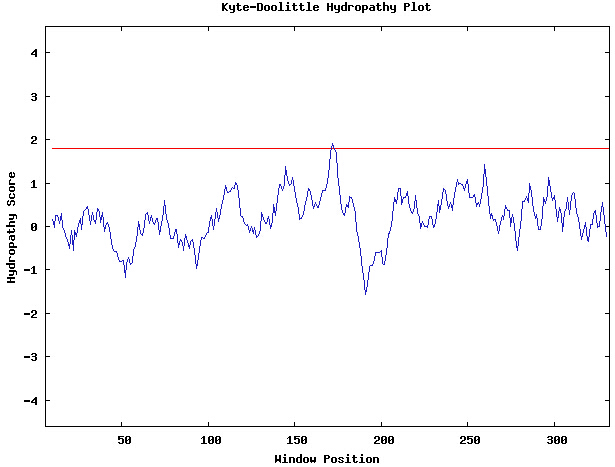
E F


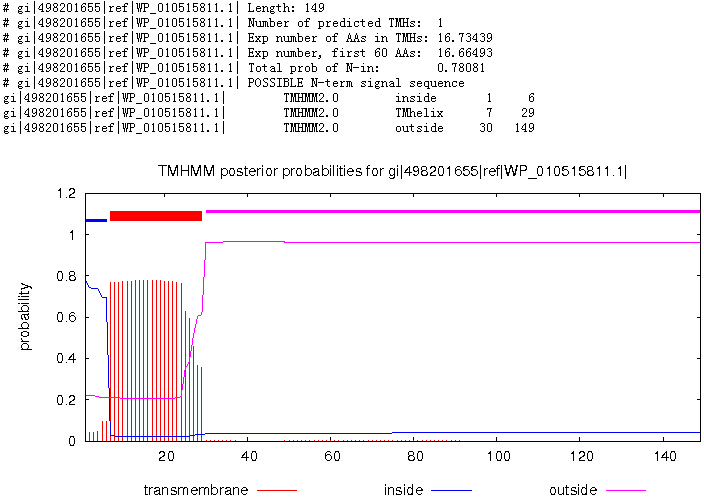


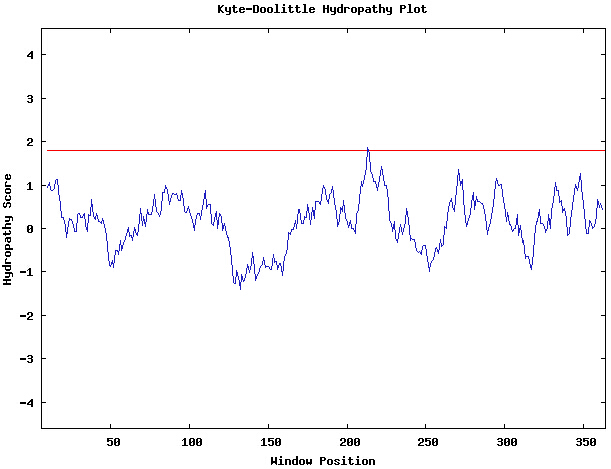
G H


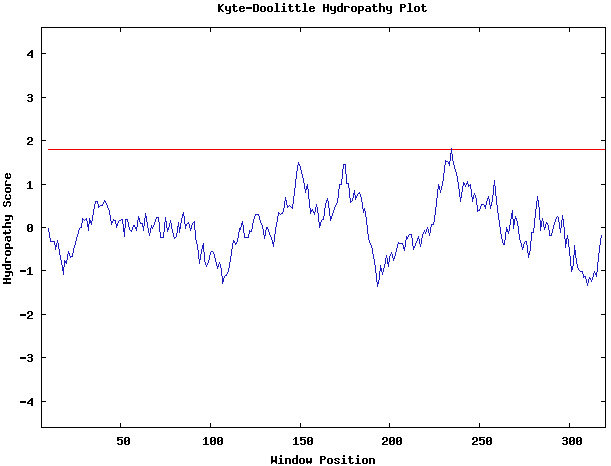

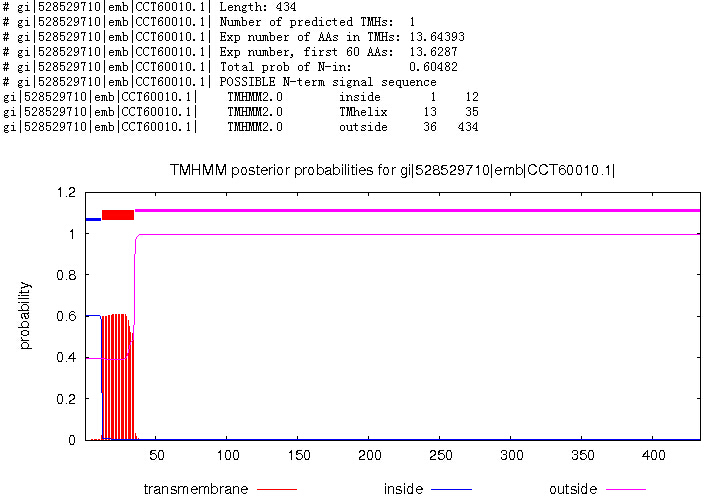


I J


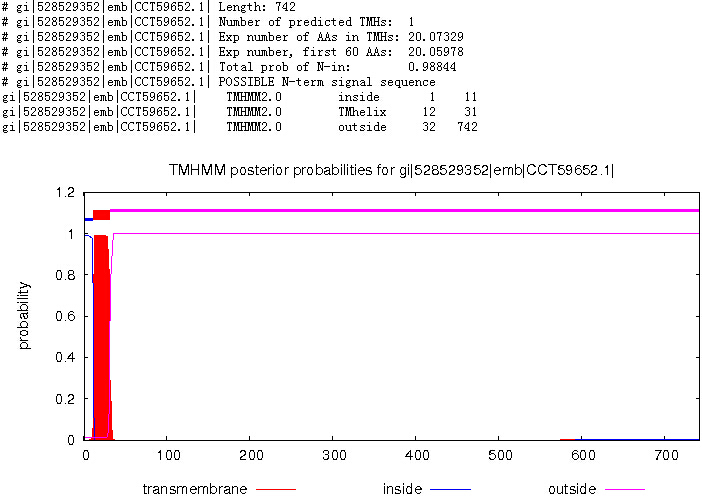

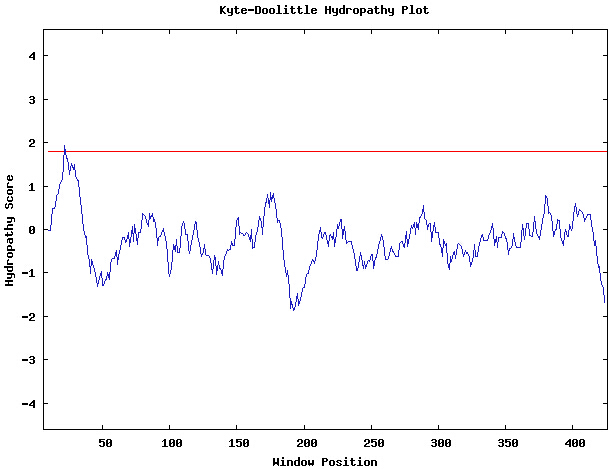
K L


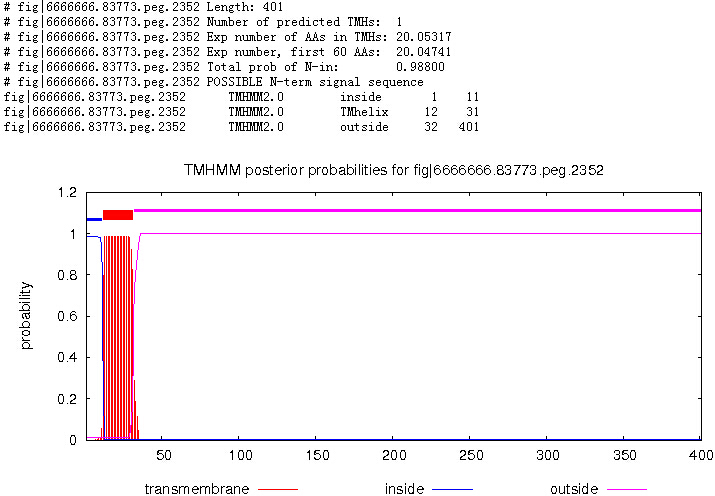

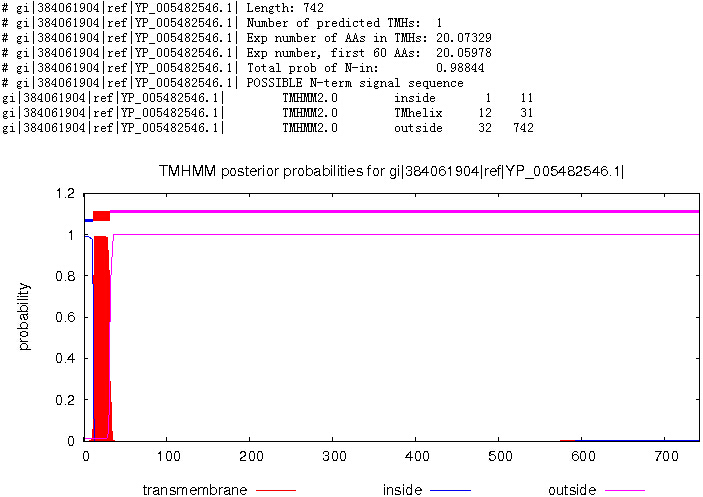


M N


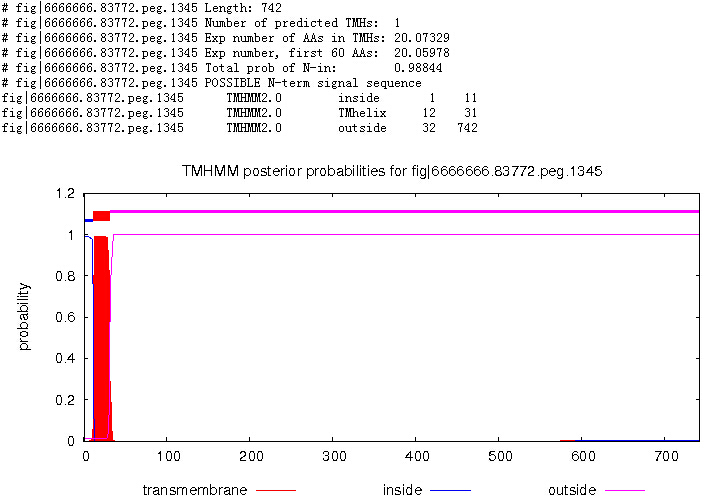


O

Figure S3 | The predicted topologies of ADHs in the AABs. A (WP_010511043.1), B (WP_019092391.1), C (WP_010508213.1), D (WP_010507548.1), E (WP_026019657.1), F (WP_026019629.1) and G (WP_019091327.1) represent the topologies of ADHs in *K. europeaus* 5P3; H (WP_010515811.1) and I (WP_010514639.1) represent the topologies of ADHs in *K. oboediens* 174Bp2; J (CCT60010.1) and K (CCT59652.1) represent the topologies of ADHs in *A. pasterianus* 386B; L (YP_005482546.1) and M (YP_004869763.1) represent the topologies of ADHs in *A. pasterianus* IFO 3283-32; N (peg.2352) represents the topology of ADHs in *A. pasterianus* CICC 20001; O (peg.1345) represents the topology of ADHs in *A. pasterianus* CGMCC 1.41. C, D, E, F, G, I and L are predicted by Kyte-Doolittle, while A, B, H, J, K, M, N and O are predicted by TMHMM 2.0.


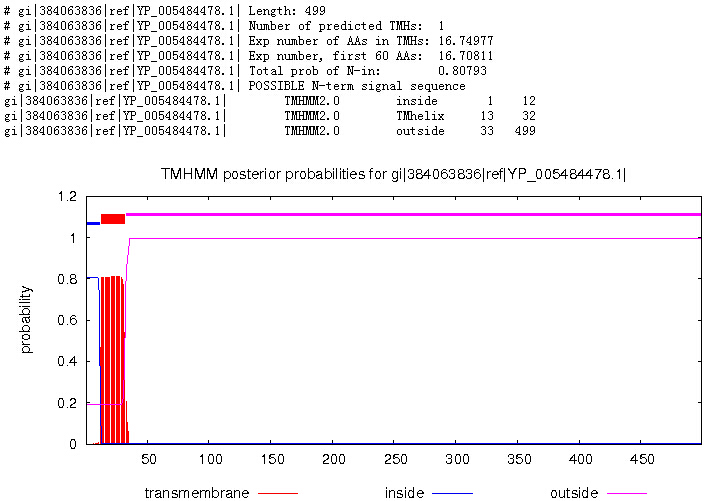

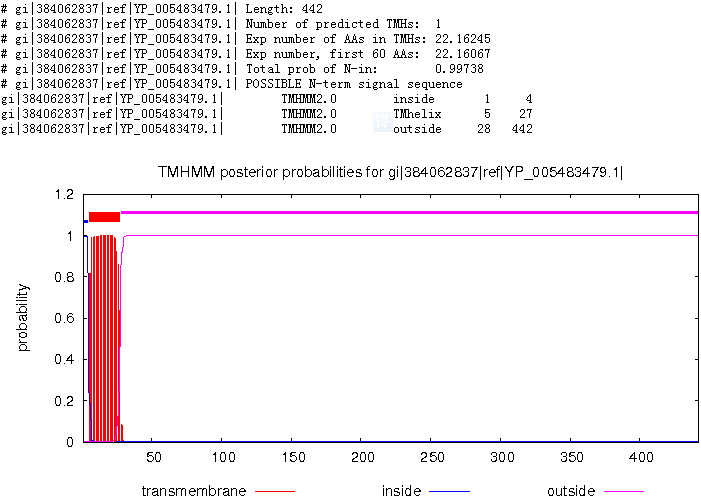
A B


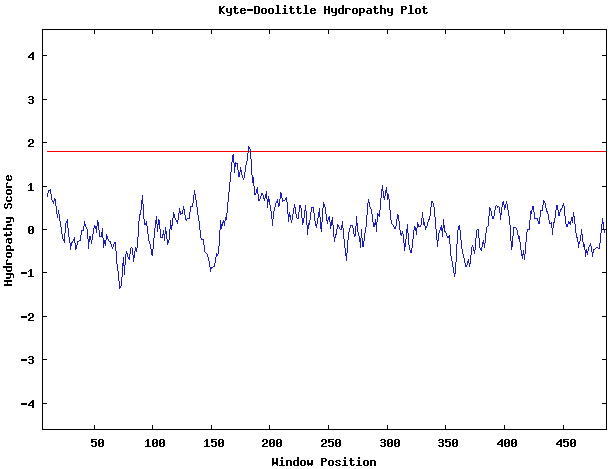

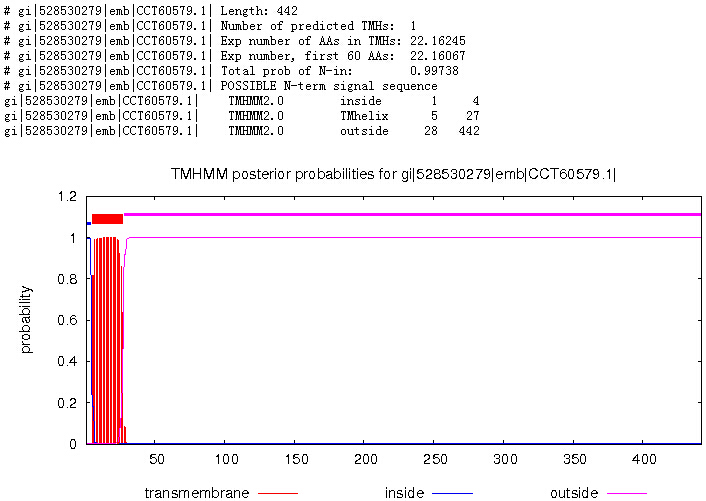


C D


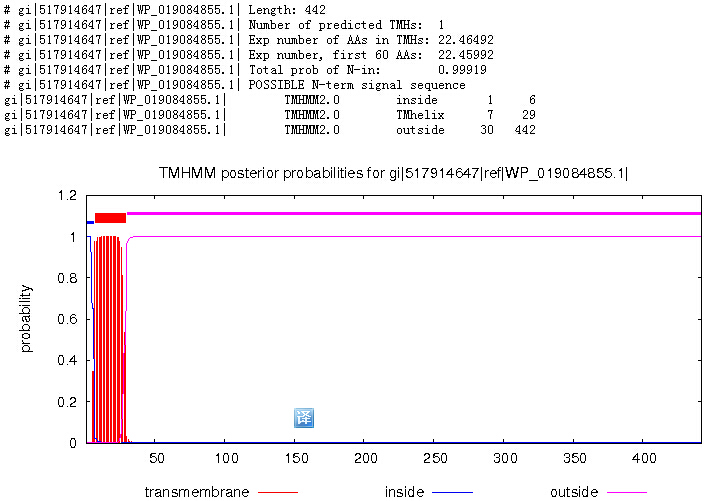

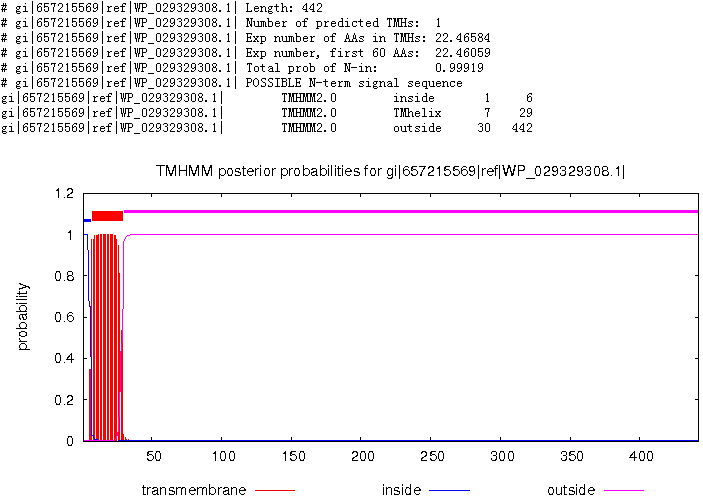


E F

Figure S4 | The predicted topologies of ALDHs in the AABs. A (YP_005484478.1), B (YP_005483479.1) and C (YP_005484624.1) represent the topology of ALDHs in *A. pasteurianus* IFO 3283-32. D (CCT60579.1), E (WP_019084855.1) and F (WP_029329308.1) represent the topology of ALDHs in *A. pasterianus* 386B, *K. europeaus* 5P3 and *K. oboediens* 174Bp2, respectively. C is predicted by Kyte-Doolittle, while A, B, D, E and F are predicted by TMHMM 2.0.

Supplementary Data

Data S1 | The number of ADHs predicted by three methods in AABs. Transmembrane topologies of all ADHs in AABs predicted using Tmpred, TMHMM and Kyte-doolittle.

| AABs | Locus | Tmpred | TMHMM | Kyte-doolittle | Conclusion ^a^ |
| --- | --- | --- | --- | --- | --- |
| *Ke*  (7/20 hits) ^b^ | WP_019092391.1 | + | + | + | + |
|  | WP_026019850.1 | - | - | + | - |
|  | WP_026018673.1 | - | - | - | - |
|  | WP_010507548.1 | + | - | + | + |
|  | WP_019091808.1 | + | - | - | - |
|  | WP_010508057.1 | + | - | - | - |
|  | WP_019091387.1 | - | - | - | - |
|  | WP_019091327.1 | + | - | + | + |
|  | WP_010509603.1 | - | - | - | - |
|  | WP_010511043.1 | + | - | - | - |
|  | WP_026019657.1 | + | - | + | + |
|  | WP_010507980.1 | + | - | - | - |
|  | WP_019091030.1 | - | - | - | - |
|  | WP_026019629.1 | + | - | + | + |
|  | WP_019091041.1 | - | - | - | - |
|  | WP_010508213.1 | + | - | + | + |
|  | WP_010508213.1 | + | - | + | + |
|  | WP_026019545.1 | + | - | - | - |
|  | WP_026019546.1 | + | - | - | - |
|  | WP_019090458.1 | - | - | - | - |
| *Ko*  (2/14 hits) | WP_010516896.1 | - | - | - | - |
|  | WP_010516306.1 | + | - | - | - |
|  | WP_010515811.1 | + | + | + | + |
|  | WP_010515418.1 | - | - | - | - |
|  | WP_010515323.1 | - | - | - | - |
|  | WP_029329553.1 | + | - | - | - |
|  | WP_010514639.1 | + | - | + | + |
|  | WP_029329445.1 | + | - | - | - |
|  | WP_029329446.1 | + | - | - | - |
|  | WP_010514275.1 | + | - | - | - |
|  | WP_010514200.1 | + | - | - | - |
|  | WP_010514001.1 | + | - | - | - |
|  | WP_010513273.1 | - | - | - | - |
|  | WP_010513157.1 | + | - | - | - |
| *Ap*_1_  (2/8 hits) | CCT58329.1 | - | - | - | - |
|  | CCT58612.1 | - | - | - | - |
|  | CCT59167.1 | + | - | - | - |
|  | CCT59588.1 | + | - | - | - |
|  | CCT59651.1 | + | - | - | - |
|  | CCT59652.1 | + | + | + | + |
|  | CCT60010.1 | + | + | + | + |
|  | CCT60256.1 | + | - | - | - |
| *Ap*_2_  (2/13 hits) | YP_005482484.1 | + | - | - | - |
|  | YP_005482545.1 | + | - | - | - |
|  | YP_005482546.1 | + | + | + | + |
|  | YP_005482779.1 | + | - | - | - |
|  | YP_005482918.1 | + | - | + | + |
|  | YP_005483157.1 | + | - | - | - |
|  | YP_005483308.1 | + | - | - | - |
|  | YP_005483718.1 | - | - | - | - |
|  | YP_005483838.1 | - | - | - | - |
|  | YP_005484002.1 | - | - | - | - |
|  | YP_005484625.1 | + | - | - | - |
|  | YP_005484736.1 | - | - | - | - |
|  | YP_005484887.1 | - | - | - | - |
| *Ap*_3_  (1/10 hits) | HN.2430 | + | - | - | - |
|  | HN.2353 | - | - | - | - |
|  | HN.2352 | + | + | + | + |
|  | HN.2119 | + | - | - | - |
|  | HN.1656 | + | - | - | - |
|  | HN.1518 | - | - | - | - |
|  | HN.873 | - | - | - | - |
|  | HN.872 | - | - | - | - |
|  | HN.609 | - | - | - | - |
|  | HN.2734 | + | - | - | - |
| *Ap*_4_  (1/8 hits) | AS.2899 | + | - | - | - |
|  | AS.2801 | - | - | - | - |
|  | AS.2227 | - | - | - | - |
|  | AS.2103 | + | - | - | - |
|  | AS.1568 | + | - | - | - |
|  | AS.1345 | + | + | + | + |
|  | AS.1279 | + | - | - | - |
|  | AS.170 | + | - | - | - |

Symbols: +, transmembrane topology predicted by one of the methods; -, non-transmembrane topology predicted by one of the methods.

Abbreviations: *Ke*, *K. europaeus* 5P3; *Ko*, *K. oboediens* 174Bp2; *Ap*_1_, *A. pasteurianus* 386B; *Ap*_2_, *A. pasteurianus* IFO 3283-32; *Ap*_3_, *A.pasteurianus* CICC 20001; *Ap*_4_, *A. pasteurianus* CGMCC 1.41.

^a^ Depending on prediction results from the three methods.

^b^ Total 20 ADHs containing 7 membrane ADHs.

Data S2 | The number of ALDHs predicted by three methods in AABs. Transmembrane topologies of all ADHs in AABs predicted using Tmpred, TMHMM and Kyte-doolittle.

| AAB | Locus | Tmpred | TMHMM | Kyte-doolittle | Conclusion ^a^ |
| --- | --- | --- | --- | --- | --- |
| *Ke*  (1/7 hits)^b^ | WP_026019896.1 | - | - | - | - |
|  | WP_019086799.1 | - | - | - | - |
|  | WP_026019798.1 | + | - | - | - |
|  | WP_019086059.1 | + | - | - | - |
|  | WP_010507111.1 | - | - | - | - |
|  | WP_019091244.1 | + | - | - | - |
|  | WP_019084855.1 | + | + | + | + |
| *Ko*  (1/5 hits) | WP_029329732.1 | - | - | - | - |
|  | WP_010516038.1 | - | - | - | - |
|  | WP_010513934.1 | - | - | - | - |
|  | WP_010513473.1 | + | - | - | - |
|  | WP_029329308.1 | + | + | + | + |
| *Ap*_1_  (1/7 hits) | CCT58273.1 | - | - | - | - |
|  | CCT59011.1 | - | - | - | - |
|  | CCT59306.1 | + | - | - | - |
|  | CCT60012.1 | - | - | - | - |
|  | CCT60125.1 | + | - | - | - |
|  | CCT60578.1 | - | - | - | - |
|  | CCT60579.1 | + | + | - | + |
| *Ap*_2_  (3/14 hits) | YP_005482483.1 | - | - | - | - |
|  | YP_005482919.1 | - | - | - | - |
|  | YP_005482920.1 | - | - | - | - |
|  | YP_005483265.1 | - | - | - | - |
|  | YP_005483477.1 | - | - | - | - |
|  | YP_005483478.1 | - | - | - | - |
|  | YP_005483479.1 | + | + | - | + |
|  | YP_005483763.1 | - | - | - | - |
|  | YP_005484467.1 | - | - | - | - |
|  | YP_005484478.1 | + | + | - | + |
|  | YP_005484624.1 | + | - | + | + |
|  | YP_005484636.1 | + | - | - | - |
|  | YP_005484655.1 | - | - | - | - |
|  | YP_005484790.1 | + | - | - | - |
| *Ap*_4_  (0/10 hits) | HN. 49 | - | - | - | - |
|  | HN. 50 | - | - | - | - |
|  | HN. 51 | - | - | - | - |
|  | HN. 1526 | - | - | - | - |
|  | HN. 1809 | - | - | - | - |
|  | HN. 1810 | + | - | - | - |
|  | HN. 1811 | - | - | - | - |
|  | HN. 1956 | - | - | - | - |
|  | HN. 1957 | - | - | - | - |
|  | HN. 2985 | - | - | - | - |
| *Ap*_5_  (0/9 hits) | AS. 5 | - | - | - | - |
|  | AS.603 | - | - | - | - |
|  | AS. 1736 | - | - | - | - |
|  | AS. 1737 | - | - | - | - |
|  | AS. 2219 | - | - | - | - |
|  | AS. 2849 | - | - | - | - |
|  | AS. 2850 | - | - | - | - |
|  | AS. 3011 | - | - | - | - |
|  | AS. 3012 | - | - | - | - |

Symbols: +, transmembrane topology predicted by one of the methods; -, non-transmembrane topology predicted by one of the methods.

Abbreviations: *Ke*, *K. europaeus* 5P3; *Ko*, *K. oboediens* 174Bp2; *Ap*_1_, *A. pasteurianus* 386B; *Ap*_2_, *A. pasteurianus* IFO 3283-32; *Ap*_3_, *A.pasteurianus* CICC 20001; *Ap*_4_, *A. pasteurianus* CGMCC 1.41.

^a^ Depending on prediction results from the three methods.

^b^ Total 7 ALDHs containing 1 membrane ALDHs

Data S3 | The number of genes related to acetic acid resistance in AABs.

| Enzymes^a^ | *Ke* | *Ko* | *Ap*_1_ | *Ap*_2_ | *Ap*_3_ | *Ap*_4_ |
| --- | --- | --- | --- | --- | --- | --- |
| Aconitate hydratase | 1 | 1 | 1 | 1 | 1 | 2 |
| Alcohol dehydrogenase | 20 | 16 | 9 | 13 | 8 | 8 |
| Aldehyde dehydrogenase | 7 | 6 | 8 | 14 | 9 | 10 |
| Acetate kinase | 1 | 1 | 1 | 1 | 1 | 1 |
| Acetyl-CoA synthetase | 2 | 2 | 1 | 2 | 5 | 3 |
| Citrate synthase | 1 | 1 | 1 | 2 | 1 | 1 |
| Phosphate acetyltransferase | 1 | 1 | 1 | 0 | 1 | 1 |
| DnaK | 4 | 4 | 3 | 4 | 1 | 1 |
| DnaJ | 4 | 4 | 7 | 7 | 4 | 4 |
| GroEL | 2 | 2 | 1 | 2 | 1 | 1 |
| GroES | 1 | 1 | 1 | 2 | 1 | 1 |
| GrpE | 1 | 1 | 2 | 2 | 1 | 1 |
| Cyclopropane-fatty-acyl-  phospholipid synthase | 1 | 1 | 0 | 1 | 1 | 1 |
| Arginine deiminase | 1 | 0 | 0 | 0 | 0 | 0 |
| Ornithine carbamoyltransferase | 1 | 1 | 1 | 1 | 1 | 3 |
| Carbamate kinase | 0 | 1 | 0 | 0 | 0 | 0 |
| Ornithine decarboxylase | 1 | 1 | 1 | 0 | 1 | 1 |
| Lysine decarboxylase | 1 | 1 | 0 | 1 | 1 | 1 |
| ABC transpoter | 86 | 93 | 43 | 21 | 50 | 56 |
| Polyamine transpoter | 1 | 1 | 0 | 2 | 0 | 0 |
| Diaminopimelate decarboxylase | 1 | 0 | 1 | 2 | 1 | 1 |
| Urease | 0 | 0 | 7 | 7 | 7 | 7 |

Abbreviations: *Ke*, *K. europaeus* 5P3; *Ko*, *K.* *oboediens* 174Bp2; *Ap*_1_, *A. pasteurianus* 386B; *Ap*_2_, *A. pasteurianus* IFO 3283-32; *Ap*_3_, *A.pasteurianus* CICC 20001; *Ap*_4_, *A. pasteurianus* CGMCC 1.41.

^a^ Enzymes related to acetic acid resistance.

Data S4 | Prediction of LTR in *Ap* species.

| *Ap* strains | Start | End | Length | LTR length | | Chr or plasm |
| --- | --- | --- | --- | --- | --- | --- |
| *Ap IFO* 3283-01 | 2589605 | 2599987 | 10383 | 1269 | | Chr |
| *Ap IFO* 3283-01-42C | 2497351 | 2507733 | 10383 | 1269 | | Chr |
| *Ap IFO* 3283-03 | 2590812 | 2601194 | 10383 | 1269 | | Chr |
| *Ap IFO* 3283-07 | 2588154 | 2598536 | 10383 | 1269 | | Chr |
| *Ap IFO* 3283-12 | 2588149 | 2598531 | 10383 | 1269 | | Chr |
| *Ap IFO* 3283-22 | 2590792 | 2601174 | 10383 | 1269 | | Chr |
| *Ap IFO* 3283-26 | 2590834 | 2601216 | 10383 | 1269 | | Chr |
| *Ap IFO* 3283-32 | 2588167 | 2598549 | 10383 | 1269 | | Chr |
| *Ap* 386B | No | | | | | |
| *Ap* CICC 20001 | No | | | | | |
| *Ap* CGMCC 1.41 | 76190 | 79365 | 3176 | | 833 | Plasm |

Abbreviations: *Ap*, *Acetobacter pasteurianus*; Chr, chromosome; Plasm, plasmid.

Data S5 | Prediction of CRISPER in *Ap* species.

| ***Ap* strains** | **Position** | **Spacers** | **length** | **DR consensus** |
| --- | --- | --- | --- | --- |
| *Ap* CICC 20001 | Chr | 1 | 90 | TTGCACAAAAACCCCC  GATTTCGACACTTTT |
|  |  | 1 | 97 | TCCCACACACCGCC  TTTAGTGGTATC |
|  | Plasm scaffold28 | 5 | 334 | CGGTTAAACCCCGCAG  ACGCGGGGAAGACT |
|  |  | 25 | 1527 | CCAGACCGCCGCATAG  GCGGTTTAGAAA |
|  |  | 1 | 89 | AAGTGTGAACTTTT  GAAGCAGTC |
| *Ap* CGMCC 1.41 | Chr | 1 | 79 | ACAACAGTCAATG  CTGGTGGTGA |
| *Ap* IFO 3283-01 | Plasm | 23 | 1431 | GTGTTCCCCGCACAC  GCGGGGATGAACCG |
| *Ap* IFO 3283-01-42 C | Plasm | 23 | 1431 | GTGTTCCCCGCACACG  CGGGGATGAACCG |
| *Ap* IFO 3283-03 | Plasm | 23 | 1431 | GTGTTCCCCGCACACGC  GGGGATGAACCG |
| *Ap* IFO 3283-07 | Plasm | 23 | 1431 | GTGTTCCCCGCACACG  CGGGGATGAACCG |
| *Ap* IFO 3283-12 | Plasm | 23 | 1431 | GTGTTCCCCGCACACG  CGGGGATGAACCG |
| *Ap* IFO 3283-22 | Plasm | 23 | 1431 | GTGTTCCCCGCACACG  CGGGGATGAACCG |
| *Ap* IFO 3283-26 | Plasm | 23 | 1431 | GTGTTCCCCGCACACG  CGGGGATGAACCG |
| *Ap* IFO 3283-32 | Plasm | 23 | 1431 | GTGTTCCCCGCACACG  CGGGGATGAACCG |
| *Ap* 386B | No | | | |

Abbreviations: *Ap*, *Acetobacter pasteurianus*; Chr, chromosome; Plasm, plasmid.
